# Mutations in *MINAR2* encoding membrane integral NOTCH2 associated receptor 2 cause deafness in humans and mice

**DOI:** 10.1073/pnas.2204084119

**Published:** 2022-06-21

**Authors:** Guney Bademci, María Lachgar-Ruiz, Mangesh Deokar, Mohammad Faraz Zafeer, Clemer Abad, Muzeyyen Yildirim Baylan, Neil J. Ingham, Jing Chen, Claire J. Sineni, Nirmal Vadgama, Ioannis Karakikes, Shengru Guo, Duygu Duman, Nitu Singh, Gaurav Harlalka, Shirish P. Jain, Barry Chioza, Katherina Walz, Karen P. Steel, Jamal Nasir, Mustafa Tekin

**Affiliations:** 1 Dr. John T. Macdonald Foundation Department of Human Genetics, University of Miami Miller School of Medicine, Miami, FL, 33136, USA; 2 Wolfson Centre for Age-Related Diseases, King's College London, London, SE1 1UL, UK; 3 Servicio de Genética, Hospital Universitario Ramón y Cajal, IRYCIS, Carretera de Colmenar km 9.100, 28034 Madrid, Spain; 4 Centro de InvestigaciónBiomédicaen Red de EnfermedadesRaras (CIBERER), 28034 Madrid, Spain; 5 RajarshiShahu College of Pharmacy, Malvihir, Buldana, India; 6 Oriental College of Pharmacy and Research, Oriental University, Indore, MP, India; 7 John P. Hussmann Institute for Human Genomics, University of Miami Miller School of Medicine, Miami, FL 33136, USA; 8 Department of Otorhinolaryngology, Faculty of Medicine, Dicle University, Diyarbakir, 21200 Turkey; 9 Cardiovascular Institute and Department of Cardiothoracic Surgery, Stanford University School of Medicine, Stanford, CA, 94305, USA; 10 Department of Audiology, Faculty of Health Sciences, Ankara University, Ankara, 06100, Turkey; 11 RILD Building, Wellcome Wolfson Centre, University of Exeter Medical School, Exeter, UK; 12 Molecular Biosciences Research Group, Faculty of Health and Society, University of Northampton, UK; 13 Department of Otolaryngology, University of Miami Miller School of Medicine, Miami, FL, 33136, USA

**Keywords:** Autosomal recessive, deafness, hearing loss, *MINAR2*, NOTCH2, Biological Sciences (Major), Genetics (Minor)

## Abstract

Discovery of deafness genes and elucidating their functions have substantially contributed to our understanding of hearing physiology and its pathologies. Here we report on DNA variants in *MINAR2,* encoding membrane integral NOTCH2-associated receptor 2, in four families underlying autosomal recessive non-syndromic deafness. Neurologic evaluation of affected individuals at ages ranging from 4 to 80 years old does not show additional abnormalities. *MINAR2* is a recently annotated gene with limited functional understanding. We detected three *MINAR2* variants, c.144G>A (p.Trp48*), c.412_419delCGGTTTTG (p.Arg138Valfs*10), and c.393G>T, in 13 individuals with congenital or prelingual-onset severe to profound sensorineural hearing loss. The c.393G>T variant is shown to disrupt a splice donor site. We show that *Minar2* is expressed in the mouse inner ear, with the protein localizing mainly in the hair cells, spiral ganglia, the spiral limbus, and the stria vascularis. Mice with loss-of-function of the Minar2 protein (*Minar2^tm1b/tm1b^*) present with rapidly progressive sensorineural hearing loss associated with a reduction in outer hair cell stereocilia in the shortest row and degeneration of hair cells at a later age. We conclude that MINAR2 is essential for hearing in humans and mice and its disruption leads to sensorineural hearing loss. Progressive hearing loss observed in mice and in some affected individuals and as well as relative preservation of hair cells provides an opportunity to interfere with hearing loss using genetic therapies.

## Introduction

Hearing loss (HL) is one of the most common sensory deficits, affecting ~1 in 500 newborns (1). Genetic factors are implicated in the majority of cases, with more than 80% of the inherited form exhibiting autosomal recessive transmission (2). No additional findings are present in over 70% of the cases, which are then classified as non-syndromic HL (Hereditary Hearing Loss Homepage, https://hereditaryhearingloss.org/) (2, 3). Genetic testing for etiologic evaluation has become a standard of care in people with congenital or childhood-onset sensorineural HL, which is caused by pathologies of the inner ear and auditory nerve (4, 5). Recent studies have shown that screening all recognized HL genes for variants reveals underlying cause in about half of the affected individuals, leaving a significant portion of people with HL with an unknown etiology (6–9). In the era of emerging genetic therapies for HL, finding the etiology of HL in affected individuals has become a critical task. This is especially relevant for progressive HL, as genetic therapies may potentially stop progression while cochlear hair cells are still alive (10–12).

MINAR2 (previously known as uncharacterized protein KIAA1024L and mouse gene *A730017C20Rik*) has recently been identified, and based on its structural similarity to MINAR1, named as major intrinsically disordered NOTCH2-associated receptor 2 or membrane integral NOTCH2-associated receptor 2 (13). A mutant mouse model of *Minar2* showed motor deficits similar to those seen in Parkinson disease, with no information about hearing abnormalities (13). A *Minar2* mutant mouse line, *Minar2^tm1b^*, has also recently been reported to show no auditory brainstem responses at 14 weeks old as part of a large HL screen (Mouse Genome Informatics; MGI: 2442934) (11). Functional aspects of MINAR2 and consequences of its dysfunction in humans remain unknown.

In this study, to better map the landscape of hereditary HL, we sought DNA variants underlying deafness in 13 affected individuals from four families. We identified three different *MINAR2* variants in the families co-segregating with HL. We further showed that homozygous *Minar2^tm1b^* mutant mice develop rapidly progressive HL associated with changes in outer hair cell stereocilia. Finally, via *in vitro* studies we demonstrated that *MINAR2* suppresses NOTCH2, suggesting that notch signaling might play a role in pathogenesis.

## Results

### Non-syndromic sensorineural HL is diagnosed in 13 individuals from four unrelated families

**Figure 1:**
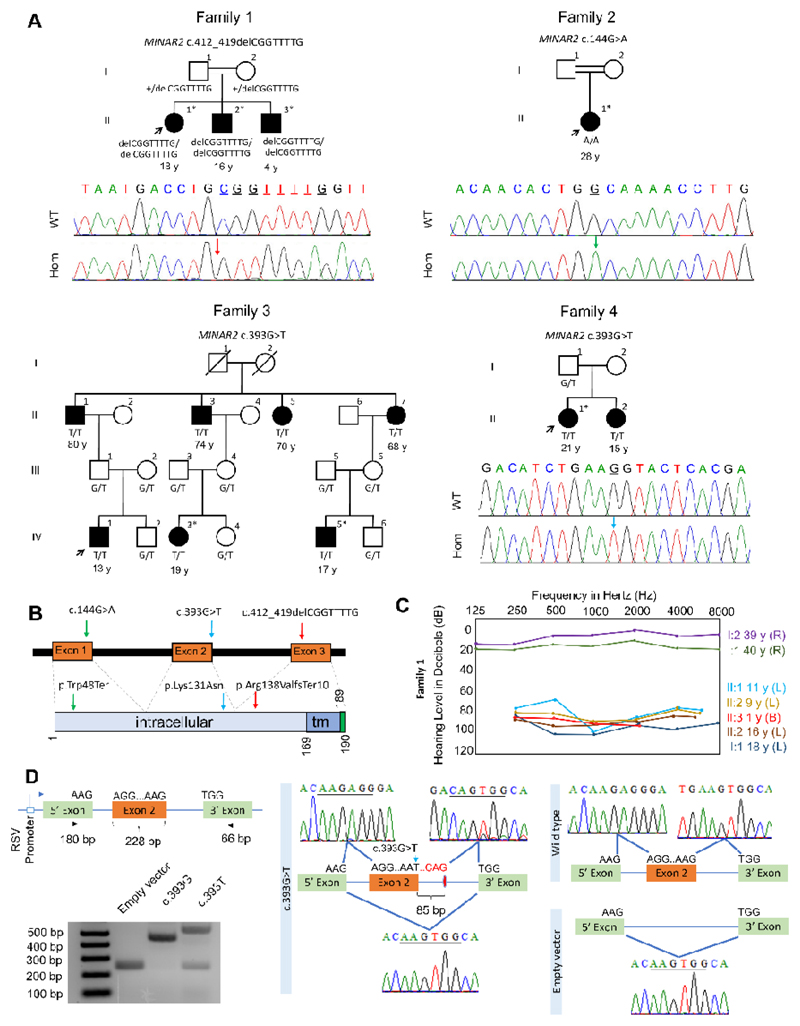
Families, *MINAR2* variants, and the effects of the c.393G>T variant on splicing. **(A)** Pedigrees and segregation of the *MINAR2* variants in families. Filled symbols denote affected individuals and double lines indicate first cousin consanguinity. Electropherograms show the identified variant. The wildtype traces are from an unrelated individual. WT: Wildtype, Hom: Homozygous mutant; exome/genome sequencing was performed in individuals marked with an (*) asterisk. **(B)** Locations of the identified variants. tm: transmembrane **(C)** Audiogram of the Family 1 (R: Right ear, L: Left ear, B: Bilateral). **(D)** *MINAR2* exon 2 inserted into a vector consisting of 5’ and 3’ exons in the exon trap assay is shown. There are larger and smaller PCR products in the c.393G>T sample compared to wildtype. Sanger sequencing confirms insertion of 85 bp at the donor site of exon 2 and skipping of exon 2.

A summary of the auditory phenotype is shown in SI Appendix, Table S1. Ages ranged from 4 to 80 years old at the last examination. Each affected individual was diagnosed with HL either at birth or during infancy. Families 1 and 2 were of Turkish ancestry (Fig. 1). Parents of Family 1 stated that in II:1 and II:2, HL was milder in younger ages and progressed to severe or profound degree by around age 10. Otoacoustic emissions were absent in these individuals. These siblings received unilateral cochlear implants at ages 12 and 10, respectively, which improved their oral communication. Individual II:3 in Family 1 was diagnosed with profound sensorineural HL after failing the newborn hearing screening test. Otoacoustic emissions were absent at diagnosis. He received a unilateral cochlear implant at age 1 and communicates orally. Pure tone audiograms in parents showed normal thresholds (SI Appendix, Fig. S1).

Families 3 and 4 were of Indian ancestry. While there is no known consanguinity in any of the marriages in these two pedigrees, they are all from the same small town belonging to the same ethnic background, i.e Hindu (religion) and Mali (Caste). All affected individuals in Family 3 were born deaf and used signs, simple words, or sounds to be able to communicate. Severity of HL appeared to have remained the same in all the affected individuals from the beginning of life. Individuals IV:1 and IV:2 in Family 4 were diagnosed with severe to profound sensorineural HL at the age of 3 years via auditory brainstem response (ABR) studies. Parents indicated a progression in the severity of HL. Their mode of communication at that age was predominantly non-verbal.

A high-resolution temporal bone CT scan or MRI was normal in at least one affected member of each family (SI Appendix, Fig. S1). None of the affected individuals had additional clinical findings for a syndromic form of deafness. Their neurodevelopmental skills were on target except for speech delay. None of the affected individuals showed impaired balance on tandem walking and Romberg test. Neurological examination was normal except for hearing loss in six affected individuals with ages ranging from 4 to 80 years. No affected individual was noted to have bradykinesia, tremor, or rigidity similar to those seen in Parkinson disease (SI Appendix, Table S2).

### *MINAR2* variants co-segregate with autosomal recessive deafness

We performed genome sequencing in four affected individuals in Families 1 and 2 and exome sequencing in three affected individuals in Families 3 and 4 (Fig. 1A; individuals marked with an asterisk, SI Appendix, Table S3). Sequencing data in affected individuals were first analyzed for variants (SNVs: Single nucleotide variants, indels: insertions and/or deletions, and CNVs: Copy number variants) in recognized HL genes retrieved from the Hereditary Hearing Loss Homepage (https://hereditaryhearingloss.org/) (3), Online Mendelian Inheritance in Man (OMIM: http://omim.org/), University of Miami Molecular Genetics Laboratory HL gene panel, and a virtual gene panel for HL (v2.176) from PanelApp (https://www.genomicsengland.co.uk ). Minor allele frequency (MAF) thresholds of 0.005 for recessive and 0.001 for dominant variants were used. Population allele frequencies were obtained from genome aggregation database (gnomAD: http://gnomad.broadinstitute.org/) (14) and the single nucleotide polymorphism database (dbSNP: https://www.ncbi.nlm.nih.gov/projects/SNP/), as well as from our internal exome/genome database that includes > 7,000 samples from different ethnicities. American College of Medical Genetics (ACMG) and ClinGen HL expert panel guidelines were followed for variant interpretation (15, 16). This analysis did not reveal a plausible variant under any inheritance model.

After excluding variants in previously recognized deafness genes, in Family 1 we filtered shared homozygous coding and splice variants (SNVs, indels, and CNVs) in three affected siblings with a MAF of <0.005 in dbSNP, gnomAD highest sub-ethnicity, and our internal control database. This filter reveals only a *MINAR2* (GeneBank: NM_001257308.2) c.412_419delCGGTTTTG (p.Arg138Valfs*10) variant in the family. The variant is located within a 9.4 MB shared homozygous run in three siblings, which is the only homozygous region >1 MB (SI Appendix, Tables S4 and S5). This frame-shift variant is predicted to introduce a premature stop codon and lead to early truncation of MINAR2.

In the proband of Family 2, *MINAR2* is located within the second longest homozygous run at chr5:128,253,080-141,730,596 (hg19) (SI Appendix, Table S6). She is homozygous for the c.144G>A (p.Trp48*) variant in *MINAR2*. This nonsense variant is predicted to cause an early stop codon and result in truncation of MINAR2.

In the exome data of two affected individuals from Family 3, filtering variants via the same criteria used in Family 1 reveals only one variant for which both individuals are homozygous: *MINAR2* c.393G>T (p.Lys131Asn). SNP arrays show that this variant is located within the only shared homozygous run, >1 MB, in all seven affected individuals in Family 3. This homozygous run is flanked by markers rs13174854 and rs377767449, which is 2.96 MB on chr5:128,738,407-131,705,915 (hg19). In Family 4, two affected siblings share a 5.76 MB homozygous run on chr5: 126,978,108-132,742,450 (hg19), flanked by markers rs11241936 and rs11242152. Exome sequencing showed that the proband in Family 4 is homozygous for the same *MINAR2* variant detected in Family 3.

None of the detected *MINAR2* variants is listed in dbSNP or gnomAD databases and all variants are predicted to be deleterious (SI Appendix, Table S7). Sanger sequencing confirmed all three *MINAR2* variants and showed that each variant co-segregates with autosomal recessive HL in all families (Fig. 1A).

### *MINAR2* c.393G>T (p.Lys131Asn) leads to aberrant splicing

While it is a missense change, the c.393G>T (p.Lys131Asn) variant substitutes the last nucleotide of exon 2 and is predicted to abolish a splice donor site (SI Appendix, Table S7 and Fig. S2). Via exon trapping experiments, we show that this variant leads to an addition of 85 intronic nucleotides into exon 2, which alters the amino acid composition of the rest of the protein leading to a premature stop codon (Fig. 1D and SI Appendix, Fig. S2). The same variant also leads to skipping of exon 2 entirely (Fig. 1D).

### *Minar2^tm1b^* homozygous mutant mice show sensorineural HL

**Figure 2:**
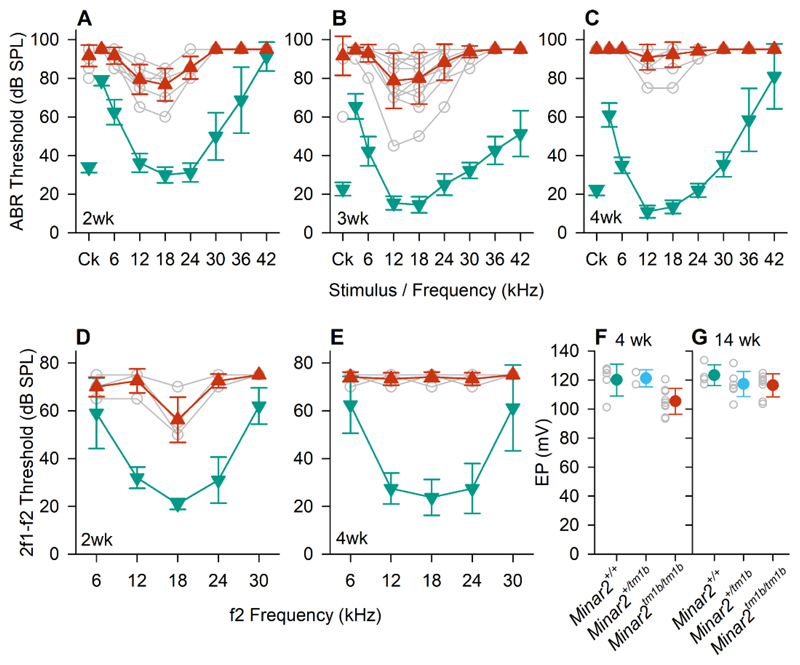
Auditory studies in *Minar2* mutant mice. **(A-C)** Auditory Brainstem Response (ABR) thresholds. Mean (±SD) ABR thresholds are plotted against stimulus for *Minar2*^+/+^ mice (teal down-triangles) and *Minar2^tm1b/tm1b^* mice (red up-triangles). Open grey circles indicate thresholds for individual *Minar2^tm1b/tm1b^* mice. Results are plotted for data obtained at P14 **(A)** (n=4 *Minar2*^+/+^, 9 *Minar2^tm1b/tm1b^*); P21. **(B)** (n=11 *Minar2*^+/+^, 12 *Minar2^tm1b/tm1b^* and P27-28 **(C) (**n=10 *Minar2*^+/+^, 11 *Minar2^tm1b/tm1b^*). Ck, click. **(D-E)** Distortion Product OtoAcoustic Emission (DPOAE) thresholds. Mean (±SD) DPOAE thresholds are plotted against f2 stimulus frequency for *Minar2* control mice (teal down-triangles) and *Minar2^tm1b/tm1b^* mice (red up-triangles). Open grey circles indicate thresholds for individual *Minar2^tm1b/tm1b^* mice. Results are plotted for data obtained at P14 **(D)** (n=4 controls, comprising 1 wildtype and 3 heterozygotes, n=4 mutants) and P27-28 **(E)** (n=4 wildtypes, n=6 mutants). **(F, G)** Endocochlear Potential (EP) in *Minar2* mutant mice aged 4 weeks (**F**) and 14 weeks old (**G**). Colored circles indicate the mean (±SD) EP measurement in *Minar2*^+/+^ (teal), *Minar2^+/tm1b^* (cyan) *Minar2^tm1b/tm1b^* (red) mice. Open grey circles indicate the EP values recorded in individual mice. At 4 weeks old (P30-32) *Minar2*^+/+^ n=5, range 101.2 – 127.8 mV; *Minar2^+/tm1b^* n=2 range 117.0 – 125.4 mV; *Minar2^tm1b/tm1b^* n=9, range 93.4 – 120.5 mV. At 14 weeks old *Minar2*^+/+^ n=4, range 117 – 133.6 mV; *Minar2^+/tm1b^* n=7, range 113.8 – 131.6 mV; *Minar2^tm1b/tm1b^* n=8, range 103.5 – 124.7 mV.

To prove causality of disruption of Minar2 in sensorineural HL, we evaluated hearing in *Minar2* mutant mice. Hearing sensitivity of *Minar2^tm1b^* mutant mice (SI Appendix, Fig. S3) was assessed using two methods: ABR, a measure of neural activity in the auditory nerve and brainstem, and Distortion Product Otoacoustic Emission (DPOAE), a measure of outer hair cell electromotility and resulting nonlinearities in the cochlea. Thresholds for both ABRs and DPOAEs were raised in homozygous mutants compared with wildtype littermates from the earliest age studied, postnatal day (P) 14, only two days after the usual onset of hearing (Fig. 2). By four weeks old, the mutant mice showed severe elevations in threshold or no response at the highest sound levels used. Endocochlear potential was only slightly reduced from a mean of 120 mV to 105 mV in *Minar2^tm1b/tm1b^* mice, with most mutant mice exhibiting an EP in the normal physiological range for mice of over 100mV (Fig. 2).

### *Minar2* is expressed in the mouse cochlea

**Figure 3:**
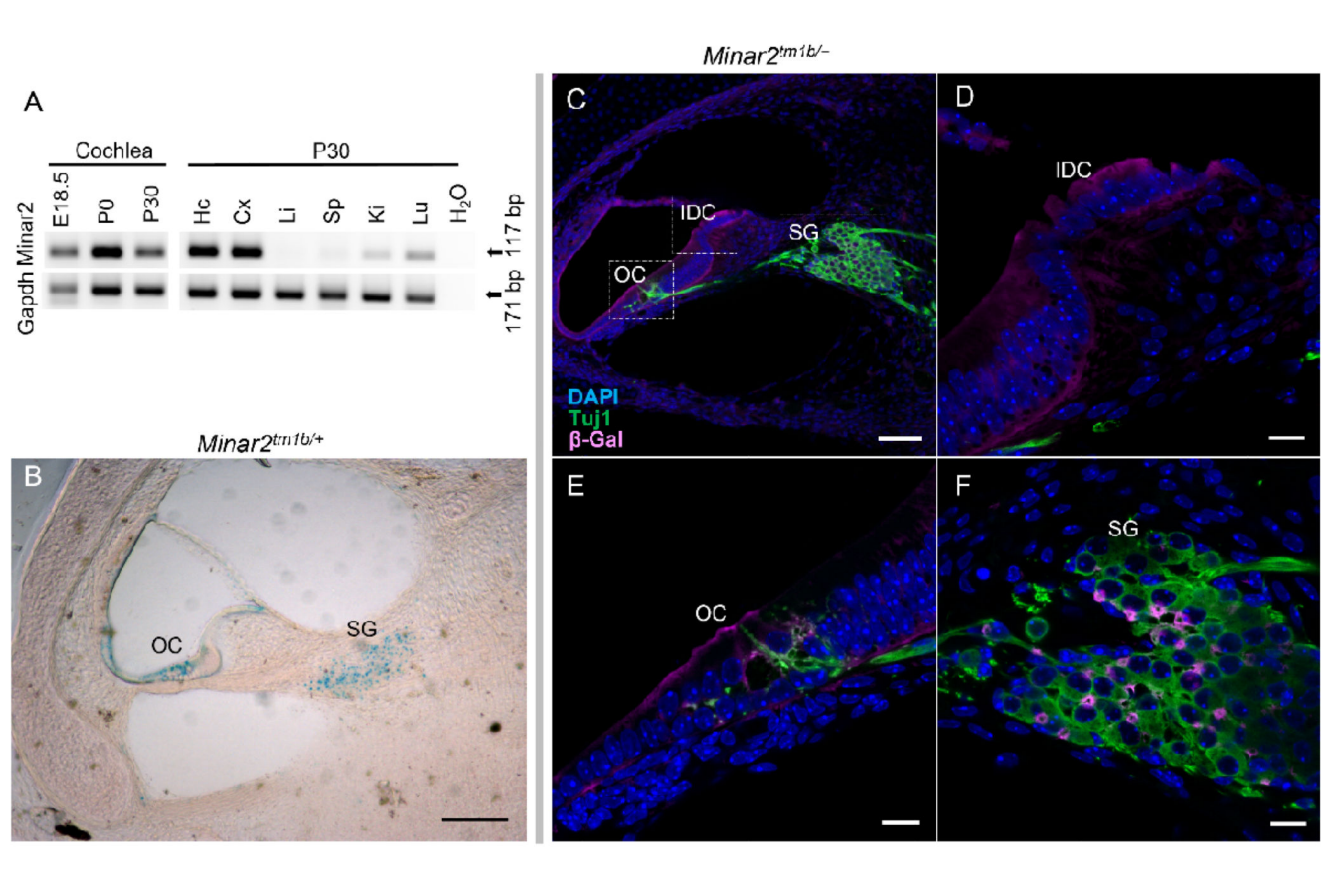
Expression of *Minar2,* localization of the protein in the cochlea and innervation of cochlear hair cells in *Minar2* heterozygous mice, using the *LacZ* reporter gene component of the inserted cassette in the mutant allele which expresses β galactosidase (see Appendix SI, Fig. S3). (A) RT-PCR of *Minar2* expression in the cochlea at embryonic 18.5 (E18.5), postnatal day 0 (P0), and day 30 (P30). Also, expression in different mouse tissues at P30 like Hc: hippocampus, Cx: cortex, Li: liver, Sp: spleen, Ki: kidney, Lu: lung. Gapdh was used as a control. (B) Localization of *Minar2* using the reporter gene LacZ of the mutant allele and in β-gal staining shown in blue. Note the localization of Minar2 at the SG: spiral ganglion, OC: organ of Corti. Scale bar: 70 µm. **(C)** Cross section from 24 kHz region of P1 mutant inner ear was labelled with anti-β gal (magenta) to detect Minar2 localization; and Tuj1 (green) to label neurons. Bar=70µm in **C** and a zoom in is shown in (**D)**, (**E)**, and (**F)** scale bar=10µm. SG; Spiral ganglion, OC: organ of Corti, IDC: Interdental cells.

To further understand the role of Minar2 in hearing, we assessed the presence of *Minar2* transcript in different mouse tissues, including the inner ear, and specifically the cochlea. Total RNA was isolated from wildtype mice at embryonic day (E)18.5, P0, and P30. RT-PCR with a forward primer located in exon 2 and a reverse primer in exon 3 of the *Minar2* gene (GenBank: NM_173759) produced a unique band of 171 bp corresponding to the wildtype mRNA in all analyzed tissues, with the exception of the liver. We find that *Minar2* is highly expressed in the inner ear, and specifically in the cochlea, at E18.5, P0, and P30 (Fig. 3A). To determine specific locations of *Minar2* expression within the cochlea, we proceeded to study *Minar2^tm1b^* heterozygous mutant mice taking advantage of the reporter gene. β-galactosidase staining of P1 cross sections and whole mount organ of Corti preparations reveals that *Minar2* is mainly localized in the hair cells, the supporting cells, spiral ganglion (including the nerve fibers), the spiral limbus, and the stria vascularis (Fig. 3B and SI Appendix, Fig. S4 A and B). Of note, the gene expression analysis resource (gEAR; https://umgear.org/ ) portal shows *Minar2* expression both in hair cells and supporting cells (SI Appendix, Fig. S4C) (17, 18). Double-labelling for Minar2 and Tuj1 in heterozygous mutant mice show that the two are expressed in different cell types, Minar2 is not present in the neurons of the spiral ganglion that were marked with Tuj1, suggesting that Minar2 is located in spiral ganglion glial cells (Fig. 3 C, E and F).

### The earliest defect in *Minar2^tm1b^* mutant mice appears in stereocilia bundles

**Figure 4:**
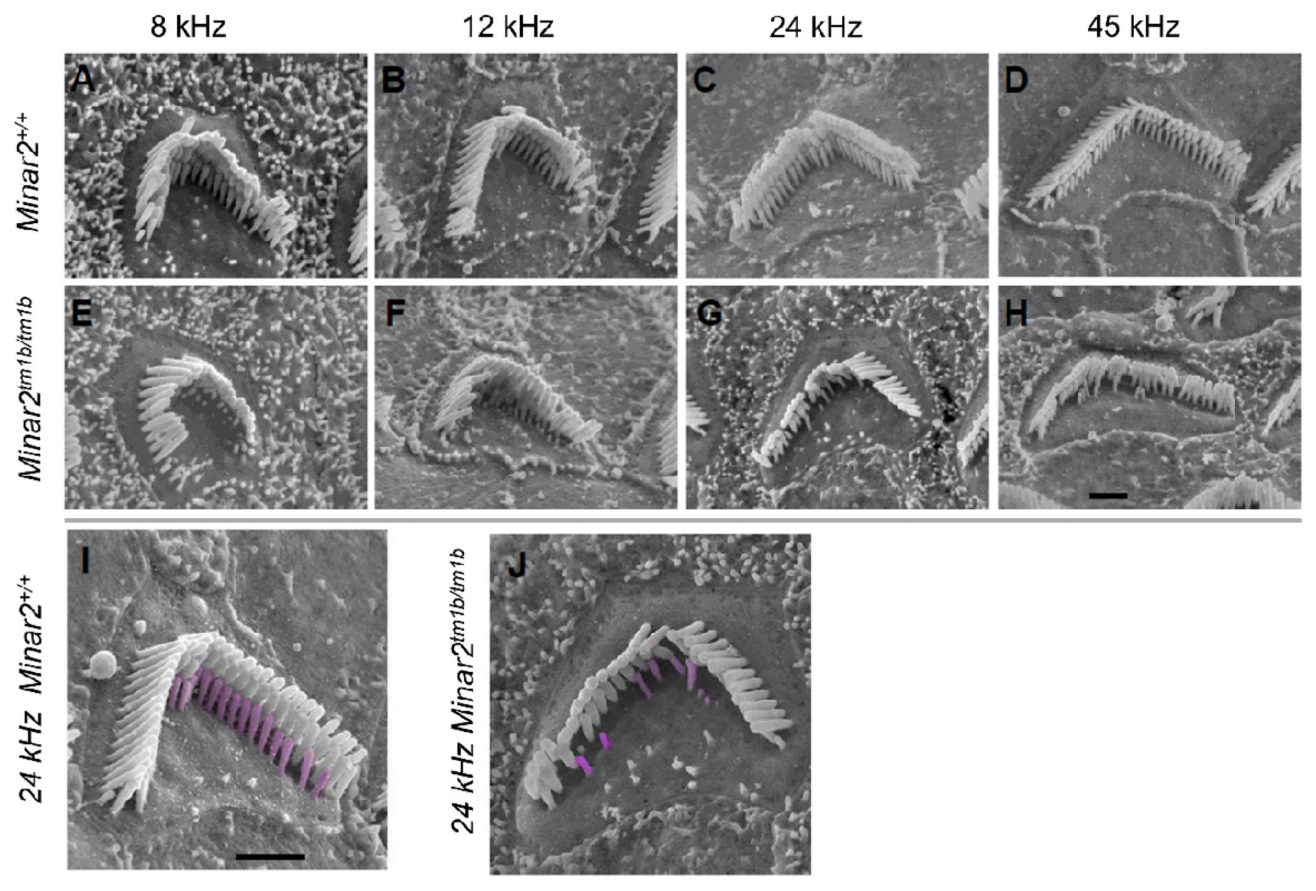
Scanning electron microscopy reveals stereocilia defects. Outer hair cells at 8kHz (85% of distance from base), 12kHz (70%), 24kHz (40%) and 45kHz (20%) best frequency locations in wildtype mice (**A-D**) and *Minar2^tm1b^* homozygotes (**E-H**). Higher magnification images with the shortest row colored in magenta in a wildtype (**I**) and a mutant (**J**) outer hair cell, showing the reduction in numbers. Scale bars (in **H** and **I**) indicate 1 μm.

**Figure 5:**
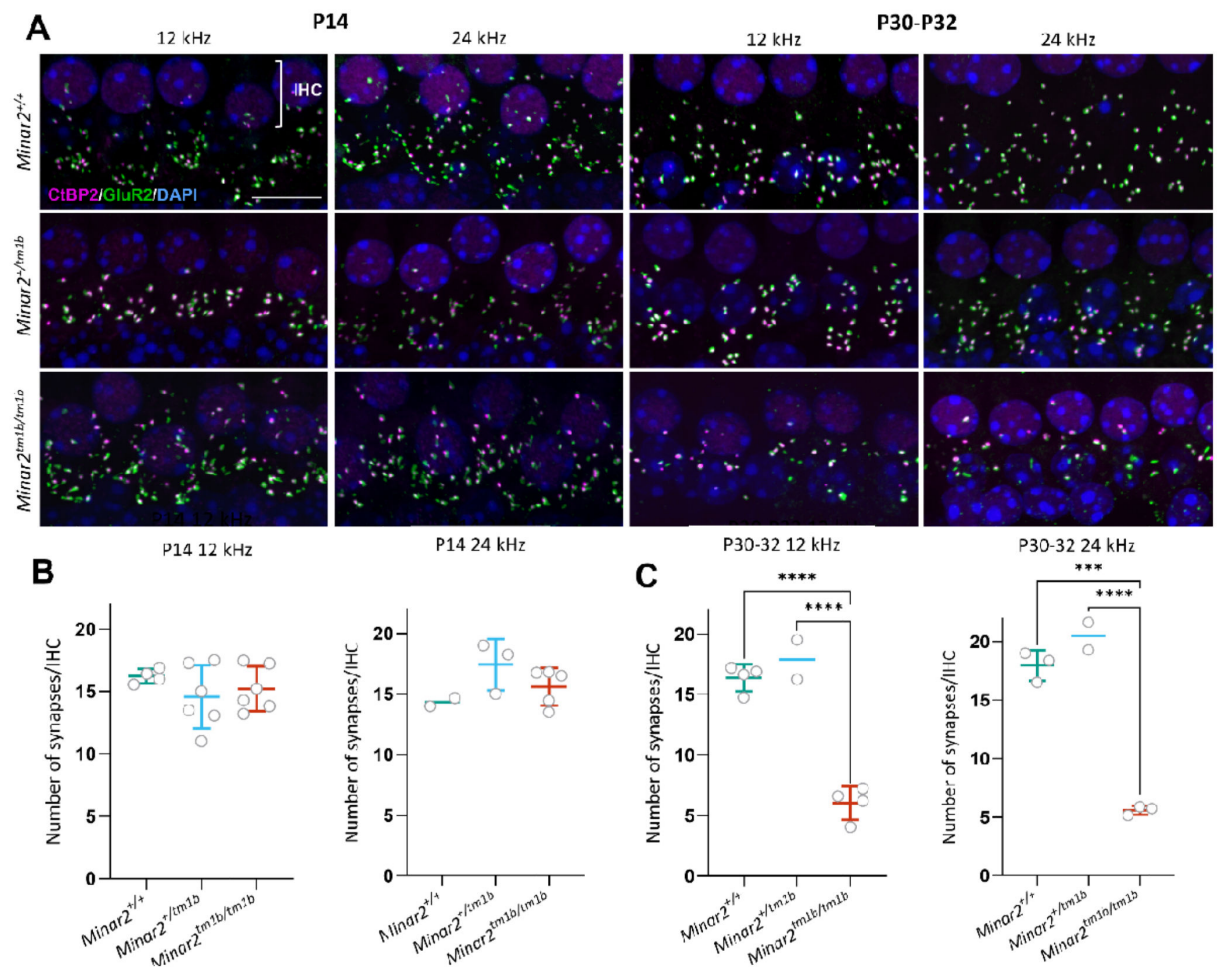
Synaptic abnormalities of cochlear hair cells in *Minar2* mutant mice. **(A)** Synapses were examined using anti-CtBP2 antibody to mark pre-synaptic ribbons (magenta) and anti-GluR2 antibody to mark postsynaptic densities (green). Nuclei are shown in blue (DAPI). Scale bar = 5 µm. The images correspond to the cochlear best-frequency regions of 12 kHz and 24 kHz. The row of nuclei at the top of each image corresponds to the IHCs. **(B, C)** Quantification of ribbon synapses per IHC at P14 **(B)** and P30-P32 **(C).** Colocalised pre and postsynaptic components were defined as a synapse. The average for each mouse is plotted as a circle. All data are shown as mean ± SD and statistically analyzed by one way ANOVA with multiple comparisons. At P14, no significant difference was seen between homozygotes (n = 6 at 12 kHz and n = 5 at 24 kHz) and heterozygotes (n = 6 at 12 kHz and n = 3 at 24 kHz) or wildtypes (n=4 at 12 kHz and n = 2 at 24 kHz). At P30-P32, there are significantly fewer colocalised synapses in *Minar2^tm1b/tm1b^* (n = 4 for 12 kHz and n = 3 for 24 kHz) compared to wildtypes (n = 4 at 12 kHz and n = 3 at 24 kHz) and compared to heterozygotes (n = 2). *** p =0.0001 and **** p < 0.0001

We used scanning electron microscopy (SEM) (Fig. 4) to examine the cochlea of mice aged 14 days old because at this age the ABR thresholds were raised but synapses appeared normal in numbers (Fig. 5). The organization of the organ of Corti appeared normal and there were very few missing stereocilia bundles in mutants, suggesting that hair cell degeneration cannot explain the raised ABR thresholds at this age (SI Appendix, Fig. S5). However, when we examined the stereocilia bundles of outer hair cells, we saw the shortest row was depleted, with reduced numbers and abnormally short stereocilia (Fig. 4). Outer hair cells along the entire length of the cochlear duct showed this feature. Inner hair cells appeared close to normal, although the middle row of stereocilia occasionally appeared more irregular than in the control littermates. Heterozygotes looked the same as wildtype littermates at this age.

### Mechanoelectrical transduction (MET) channels are functional in *Minar2^tm1b/tm1b^* mice

To study the MET, we used the FM1-43 FX dye, a molecule commonly used to assay the MET channels function, since functional MET channels are required for the uptake of this fluorescent dye into the hair cells (19). We perfused cochleae from P14 mice and observed FM1-43 FX dye uptake by both inner and outer hair cells in *Minar2^+/+^* and *Minar2^tm1b/tm1b^* mice. Heterozygotes looked the same as wildtype littermates. While the intensity of the staining varied from mouse to mouse, all the samples showed uptake of the molecule specifically in hair cells, indicating that the MET channels are active in the *Minar2* mutants (SI Appendix, Fig. S6).

### Hair cell degeneration is a secondary feature in *Minar2^tm1b^* mutant mice

**Figure 6:**
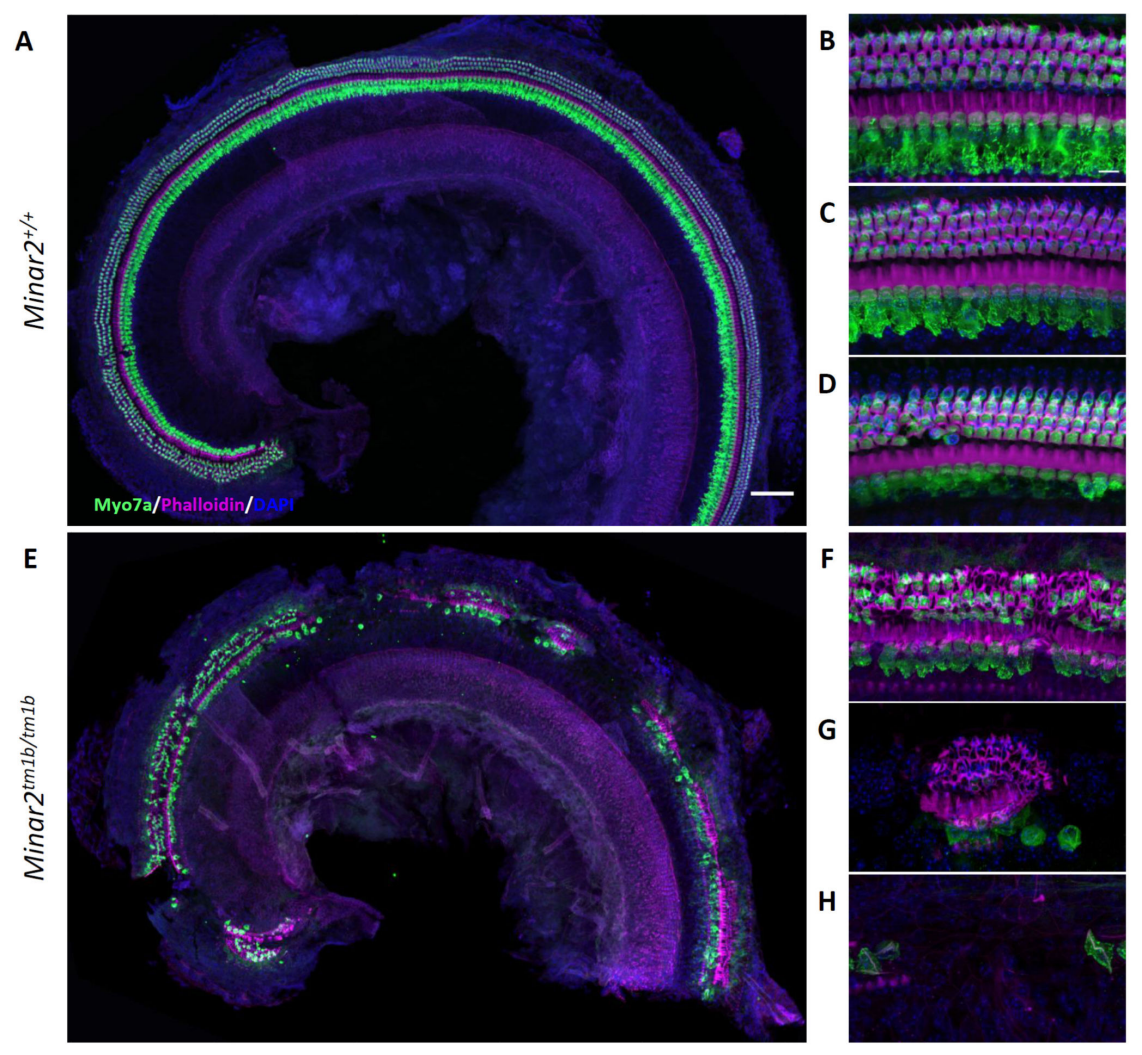
Hair cell degeneration pattern in *Minar2^tm1b^* mutant mice at 14 weeks old. Confocal maximum intensity projection images of the whole-mount organ of Corti of 14-week old mice. Hair cells were examined using anti-Myo7a antibody (green) and phalloidin (magenta). Nuclei are shown in blue (DAPI). Scale bar= 200 µm in A and 10µm in B. The images are representative examples of *Minar2*^+/+^ **(A, B, C, D)** and *Minar2^tm1b/tm1b^* **(E, F, G, H)** mice. **(A, E)** Overview of the apical half of the cochlea. **(B, C, D)** High magnification images corresponding to the 12 kHz, 24 kHz and 36 kHz best-frequency regions respectively. **(F, G, H)** High magnification images of three different patterns of hair cell loss observed in *Minar2^tm1b^* mutant mice. All 5 homozygotes that were studied at 14 weeks old showed similar patches of loss of the organ of Corti at varying locations along the length of the cochlear duct interspersed with regions that showed only scattered hair cell loss. *Minar2*^+/+^ (n = 4), *Minar2^+/tm1b^* (n = 5) and *Minar2^tm1b/tm1b^* (n = 5).

Hair cell degeneration is a common feature in HL, and extensive loss of hair cells may preclude some approaches to treatments. Therefore, we examined older *Minar2^tm1b^* mutant mice, aged 14 weeks, to assess the loss of hair cells. We have previously reported that these mutants show no auditory brainstem responses at 14 weeks old (11) and the EP is normal at this age (Fig. 2G). At earlier ages we see minimal evidence of any hair cell degeneration (SI Appendix, Fig. S5). However, by 14 weeks of age, there is extensive loss of hair cells, with some discrete patches distributed along the length of the cochlear duct where the entire organ of Corti appears to have degenerated while other regions show scattered missing hair cells, both inner and outer, within the organ of Corti (Fig. 6). Heterozygotes had a similar appearance to the wildtype mice.

### *Minar2^tm1b^* mutant mice show reduction in the number of inner hair cell ribbon synapses at P30

**Figure 7:**
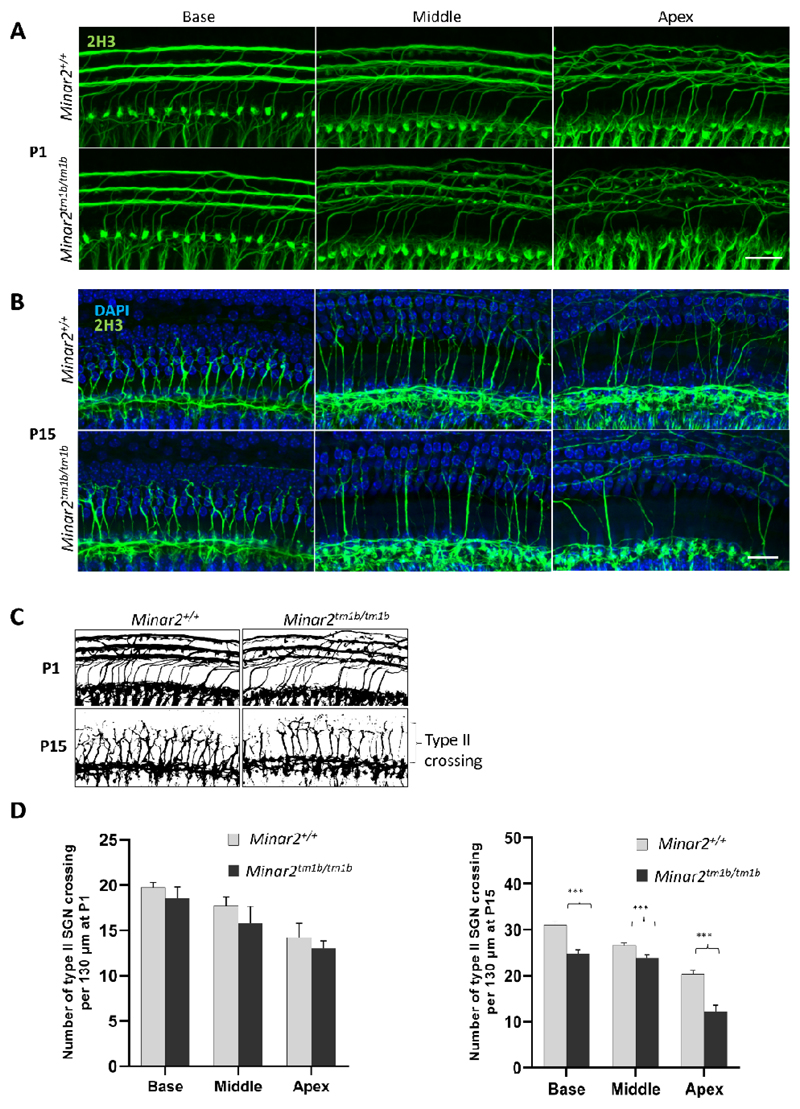
Confocal maximum intensity projection images from 24 kHz region of the whole-mount cochlea. **(A and B)** Total innervation with anti-2H3 Neurofilament label (green) for P1 and P14 (N=3 each group) wild type and mutant mice; scale bar A and B =20 µm **(C)** Schematic diagram of whole mount cochlea demonstrating the innervation pattern of type II nerves at P1 and P14 wild type and mutant mice. **(D)** The total number of crossings of type II nerves is show as histogram (n=3; *** P<0.001.)

To understand underlying pathophysiology further, we evaluated the innervation pattern of hair cells by staining neurons with an anti-Neurofilament antibody in the organ of Corti at P1 (SI Appendix, Fig. S7). Homozygous mutant mice show an altered innervation pattern of hair cells with a reduction in the nerve fibers around inner hair cells and a dispersed distribution of nerve fibers towards outer hair cells. *Minar2^tm1b^* mutant mice show reduction in the number of crossings of type II nerves at P14 (Fig. 7). We subsequently examined synapses of inner hair cells using anti-CtBP2 antibody to label presynaptic ribbons and anti-GluR2 antibody to mark the AMPA receptor subunit R2, a part of the post-synaptic density. Ribbon synapses look qualitatively normal at both ages tested, P14 and P30, and the number of synapses per inner hair cell in homozygous mutants was not significantly different from numbers in heterozygotes or wildtype controls at P14, indicating that the raised ABR thresholds observed at that age in mutants are not due to the loss of ribbon synapses (Fig. 5 A and B). However, by P30-P32 *Minar2^tm1b^* mutant mice did show a significant decrease in the number of synapses compared to wildtype mice (Fig. 5 A and C), and this may contribute to the progression of HL.

### Overexpression of *MINAR2* suppresses angiogenesis and MINAR2 variants abolish this effect

**Figure 8:**
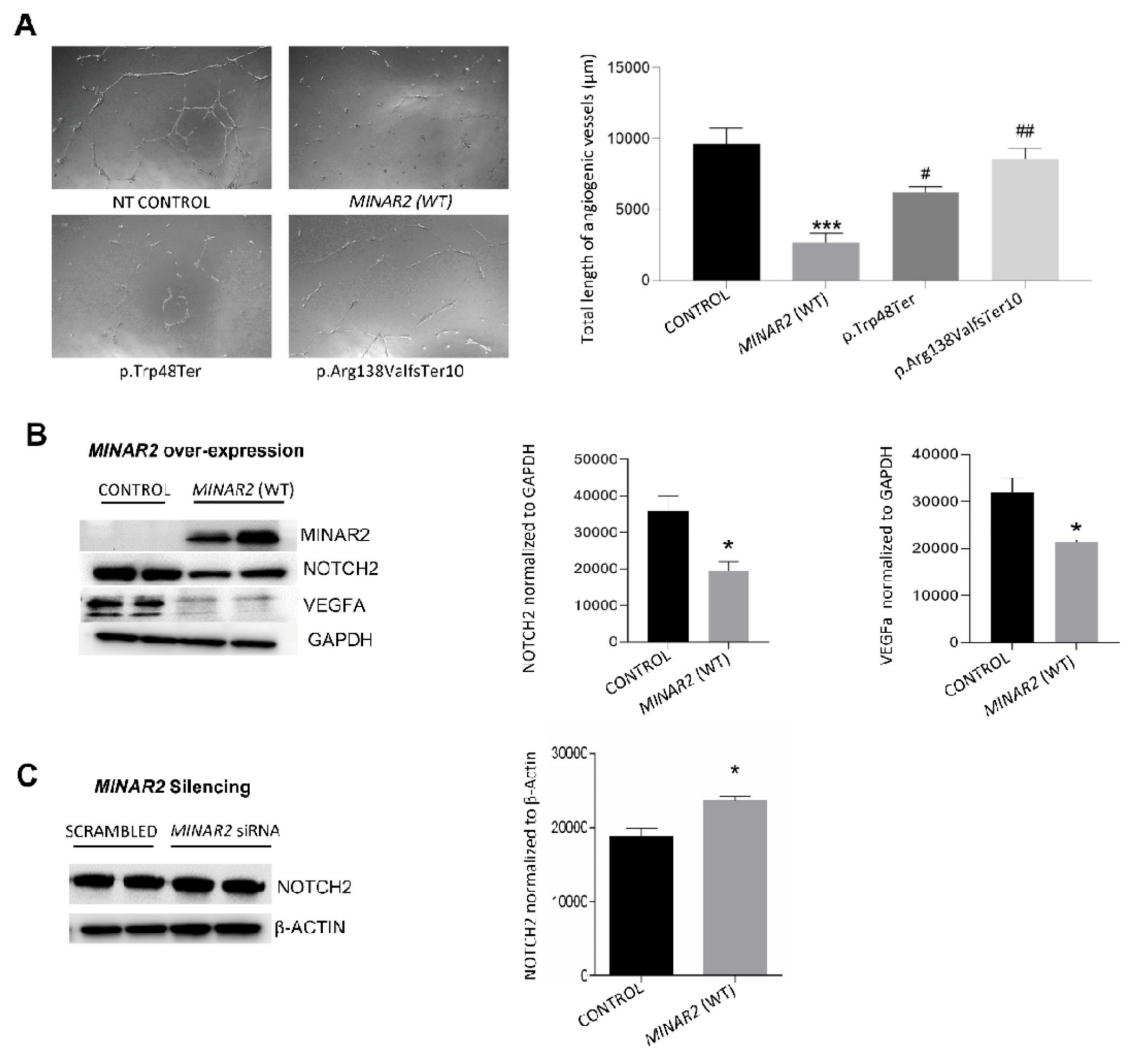
Effects of *MINAR2* on angiogenesis, NOTCH2 and VEGFA. **(A)** Angiogenic potential of non-transfected (NT) HUVEC compared to cells transfected with *MINAR2* wildtype (WT), and *MINAR2* p.Trp48* and p.Arg138Valfs*10 plasmid constructs. Right graph shows analysis of total length of angiogenic vessels formed after incubation of 12 hrs on Matrigel coated wells; images were analyzed with imageJ angioanalyzer and results are expressed as mean±SEM. Significant differences were shown as *** (p≤0.001) when compared to control and as ## (p ≤0.01) when compared to *MINAR2(WT)*. **(B)** Left: immunoblot images showing the effect of *MINAR2(WT)* transient overexpression on NOTCH2 and VEGFA protein levels in HUVEC cells; Right: statistical analysis of GAPDH-normalized relative protein expressions of NOTCH2 and VEGFA. Results are expressed as mean±SEM and significant differences are shown as *(p≤0.05) when compared to control. **(C)** Left: immunoblot images of siRNA-induced MINAR2 silencing on NOTCH2 protein levels in PC12 cells; Right: beta-actin-normalized relative protein expression of NOTCH2. Results are expressed as mean±SEM and significant differences are shown as *(p≤0.05) when compared to control. Data analyzed by one way ANOVA with multiple comparisons.

As MINAR1 has been shown to inhibit angiogenesis (20), we investigated whether MINAR2 shows the same effect on angiogenesis in human umbilical vein endothelial cells (HUVEC). These studies reveal that transient overexpression of *MINAR2* suppresses angiogenesis (Fig. 8A). In addition, overexpressing each *MINAR2* variant detected in Families 1 and 2 in HUVEC reduces suppression of angiogenesis, suggesting that they are loss-of-function variants (Fig. 8A).

### Expression levels of *MINAR2* are inversely correlated with intracellular NOTCH2 abundance

MINAR2 is named based on its structural similarity to MINAR1, which is shown to be involved in NOTCH2 signaling (20). While MINAR2 is a much smaller protein compared to MINAR1 (190 vs 917 amino acids) (20), it has recently been shown to bind NOTCH2 in a co-immunoprecipitation assay (13). Thus, we set out to explore if NOTCH2 abundance in cells is correlated with *MINAR2* expression (21, 22). Furthermore, as NOTCH and VEGF signaling pathways are known to interact during angiogenesis (23), we determined the effect of *MINAR2* expression on VEGF. Our studies show that overexpression of wildtype *MINAR2* in HUVEC is associated with reduced abundance of NOTCH2 and VEGFA (a prominent VEGF protein in vascular endothelial cells) (Fig. 8B). Silencing of *MINAR2* in PC12 cells, which endogenously express *MINAR2,* leads to an increase in NOTCH2 and confirms the suppressor effect of *MINAR2* on NOTCH2 abundance (Fig. 8C and SI Appendix, Fig. S8).

### MINAR2 is involved in MAP kinase and mTOR pathways

MINAR1 has been shown to regulate neurite outgrowth in a neuroendocrine cell line PC12 (24), in which neurite outgrowth via nerve growth factor is achieved by MAPK and PI3K pathways (25). The mTOR complex is a downstream target of PI3K, which contains mTORC1 and mTORC2 complexes in its central catalytic domain (24). We tested the effects of MINAR2 on the MAPK pathway by transiently overexpressing wildtype *MINAR2* in PC12 cells and detecting levels of ERK1/2 and pERK1/2, a crucial kinase of the MAPK signaling pathway. These studies show that overexpression of wildtype *MINAR2* reduces the abundance of pERK1/2, the active form of ERK1/2. On the other hand, overexpression of the two *MINAR2* deafness variants in Families 1 and 2 does not show this effect, again supporting their loss-of-function (SI Appendix, Fig. S9A). When we silenced *MINAR2* in PC12 cells, we observed an increase in a functional protein in mTORC1 activity, P-S6, at 12 hours (SI Appendix, Fig. S9B). However, there was no difference in pAKT, a component of mTORC2 activity (SI Appendix, Fig. S9B).

## Discussion

In this study we present three loss-of-function variants in *MINAR2* in four families co-segregating with non-syndromic sensorineural HL. Hearing loss started at birth or in early childhood in all 13 affected individuals. A progressive HL reaching to severe to profound degree during childhood was noted in four affected individuals. In addition to ABR thresholds showing sensorineural HL, otoacoustic emissions were absent in three children tested, suggesting dysfunction of both inner hair cell/acoustic nerve and outer hair cells. To support causality of the *MINAR2* mutations in deafness and to begin understanding the mechanism of HL, we show that *Minar2^tm1b/tm1b^* mice have a severe, progressive increase in ABR thresholds from two weeks old onwards (around the time that mice start to hear), with very few responses by four weeks old. Otoacoustic emissions can be detected in the mutant mice but at raised thresholds at two weeks old, and these responses are mostly absent by four weeks old, implicating outer hair cells in the pathology. In conclusion, audiological findings both in humans and mice show that loss of MINAR2 function results in early-onset and sensorineural HL that rapidly progresses to severe to profound deafness.

The reduced number of stereocilia in the shortest row on outer hair cells would be expected to have a severe impact upon the number of transduction channel complexes available and suggests that the primary defect in these mutant mice is located at the top of the hair cell. However, we have demonstrated that the remaining MET channels are active in *Minar2^tm1b/tm1b^* mice, since the hair cells of these mice are able to uptake FM1-43 dye. Degeneration of hair cells (Fig. 6) and synaptic defects (Fig. 5) occur later in development and cannot explain the raised ABR and DPOAE thresholds at 14 days old in the *Minar2^tm1b^* homozygotes. Mutations of other genes also lead to loss of the shorter stereocilia associated with hearing impairment, for example the *Baiap2l2* mutant (26) and the *Clrn2* mouse mutant (27).

The Notch pathway is a highly conserved intercellular signaling cascade that is activated by the interaction of transmembrane ligands (Delta and Jagged) with Notch receptors, which are usually expressed on the surface of neighboring cells. Binding of the Notch Ligand to receptor induces cleavage of the Notch receptors intracellular domain, which binds to multiple DNA-binding proteins in the nucleus (28–30). In the initial stages of angiogenesis, Notch activation is generally repressed to allow proliferation of endothelial cells in response to VEGF stimulation, and its expression is later upregulated when endothelial cells stop proliferating and the vessels begin to stabilize (31–37). While it is possible that the effects of MINAR2 on angiogenesis may play a role in hearing, we did not observe abnormal vascular structures in the cochlea of homozygous mutant mice. Moreover, the most sensitive part of the cochlea to vascular function is the generation of EP by the stria vascularis. In this study, EP was within the normal range in homozygous mutant mice. Our results show that MINAR2 and NOTCH2 have a regulatory relationship. In the mammalian cochlea, there is no regeneration of sensory hair cells and the strictly controlled pattern of alternating hair cells and supporting cells in the organ of Corti is set up in early development. Notch signaling is believed to be a critical part of the process of cells deciding their fate as a hair cell or a supporting cell and many genes involved in notch signaling underlie hearing impairment (38, 39). As our examination of the organ of Corti of young mice shows (SI Appendix, Fig. S5), the pattern is set up correctly, ruling out an early developmental defect of cell fate or differentiation. However, notch signally may also play a role in longer-term maintenance of the organ of Corti, and our findings of progressive deterioration of this organ resulting from mutation of a gene known to be involved in notch signaling supports this suggestion.

MINAR family proteins (MINAR1 and MINAR2) have been proposed to be associated with the inhibition of cell proliferation (13, 20). In this study, we did not observe any proliferation effect in different ages of *Minar2*^tm1b/tm1b^ mice. Terminal mitoses of hair cells occur early at E12-14 before birth (40). The confocal images illustrated in Fig. 6 all indicate loss of cells from the normal regular array, with no sign of any proliferation.

Our findings of an abnormal innervation pattern of hair cells as early as P1, as well as a significant decrease in the numbers of intact synapses at four weeks old, suggest that abnormal innervation of hair cells may contribute to the severe auditory dysfunction in *Minar2^tm1b/tm1b^* mice. Based on these findings, Minar2 may be playing a role in the maintenance of hair cell synapses. MINAR2 is structurally similar to MINAR1, which has been reported to be involved in controlling neurite formation during neuronal differentiation through DEP Domain Containing MTOR Interacting Protein (DEPTOR). In this study, we show that similar to MINAR1, MINAR2 downregulates mTOR signaling (SI Appendix, Fig. S9B), which may contribute to the abnormal innervation pattern observed in the organ of Corti of *Minar2* mutant mice.

It is notable that although the sensory hair cells are present up to four weeks old and are able to take up FM1-43 dye through transduction channels, they do not appear to be functioning normally as shown by the raised ABR and DPOAE thresholds. Most genetic and environmental causes of sensorineural HL lead to permanent loss of hair cells, reducing the chance of gene therapy or gene editing approaches. Progressive HL associated with a relatively normal appearance of hair cells in young ages makes *MINAR2* a promising target for genetic therapies. Further elucidation of its role in the maintenance of hair cell synapses and stereocilia bundles may open new avenues to treat more common forms of HL resulting from similar mechanisms.

## Materials and Methods

More details of Materials and Methods are in the SI Appendix.

### Study approval

This study was approved by the University of Miami Institutional Review Board (20081138-USA), as well as by the Ethics Committees of Ankara University Medical School (012413-Turkey) and Rajarshi Shahu College of Pharmacy (180720-India). A signed informed consent was obtained from all participants or, in the case of a minor, from the parents.

### DNA Sequencing and Bioinformatics Analysis

Genome sequencing was performed in Family 1 individuals II:1, II:2, II:3 and Family 2 II:1 (Fig. 1A and SI Appendix, Table S3) (41, 42). Reads were mapped to human genome reference (NCBI build37/hg 19 version) with Burrows Wheeler Aligner (BWA). Genome Analysis Toolkit (GATK) was used for variant calling (43–45). Copy number variants (CNVs) were called using CNVnator (46). Enlis Genome Research software (https://www.enlis.com/) was used to identify runs of homozygosity in Family 1 and Family 2 from genome sequencing data (SI Appendix, Tables S4, S5 and S6).

Illumina Infinium Global Screening Array (GSA) v2 (Illumina) kit was used for genotyping in 16 members of Family 3 and three members of Family 4 to map the shared homozygous regions in affected individuals. Additional, exome sequencing was performed on a HiSeq 2000 platform (Illumina), as described previously (47), in individuals IV:3 and IV:5 in Family 3 and IV:1 in Family 4 (SI Appendix, Table S3).

Genome sequencing data was deposited in the NCBI’s BioProject database: PRJNA623118, BioSample: SAMN27763770 (Family 1, individual II:1) and BioSample: SAMN27763769 (Family 2, individual II:1).

### Ethics statement for animals

Mouse studies in the UK were carried out in accordance with UK Home Office regulations and the UK Animals (Scientific Procedures) Act of 1986 (ASPA) under UK Home Office licenses, and the study was approved by the King's College London Ethical Review Committee. Mice were culled using methods approved under these licenses to minimize any possibility of suffering. All procedures in Miami were approved by the University of Miami Institutional Animal Care and Use Committee and followed the NIH guidelines “Using Animals in Intramural Research.”

### Mouse generation

*Minar2^tm1a^* mutant mice were generated at the Wellcome Sanger Institute by blastocyst microinjection of targeted ES cells on a C57BL/6N genetic background (48, 49). *Minar2^tm1b(KOMP)Wtsi^* mice (*Minar2^tm1b^*) were generated from *Minar2^tm1a(KOMP)Wtsi^* mice by exposure of pre-implantation embryos to soluble Cre recombinase (50) which leads to recombination between LoxP sites, removing the selection cassette and exon 2 to produce mice carrying the *Minar2^tm1b^* allele (SI Appendix, Fig. S3). Both *tm1a* and *tm1b* alleles are available via The European Mouse Mutant Archive (EMMA; https://www.infrafrontier.eu ).

### Statistics

A one-way ANOVA with multiple comparisons was used to compare groups for count of synapses in mouse inner ear and was also used for angiogenesis, MAPK, and mTOR assays.

## Supplementary Material

Supporting information

## Significance Statement

Molecular components of hearing in mammalians are not completely delineated. Via a genetic approach conducted in families with sensorineural hearing loss, this study presents MINAR2 as an indispensable element of hearing in humans. Similarly, disruption of Minar2 in mice leads to progressive hearing loss associated with alterations in the stereocilia of hair cells, the receptors of hearing, while hair cells remain intact until later in life. We present *MINAR2* as a gene working in the inner ear that is essential for hearing in humans and mice. Degeneration of sensory epithelium is a common consequence of hereditary deafness precluding genetic therapies. The preservation of hair cells in mutant mice at young ages makes MINAR2 a good candidate for intervention.
