# Supplementary material for: Mutations in MINAR2 encoding membrane integral NOTCH2 associated receptor 2 cause deafness in humans and mice
Source: Proc Natl Acad Sci U S A. Author manuscript; Available in PMC 2022 Jul 7. (PMC9245706; doi:10.1073/pnas.2204084119)
Supplement: Supporting information [file EMS146286-supplement-Supporting_information.docx]

**Supporting information**

**Table S1:** Audiological phenotype in affected individuals

| **ID** | **Age^1^(years)** | **Sex** | **Age at HL onset** | **Severity** | **Progressive HL** | **Laterality** |
| --- | --- | --- | --- | --- | --- | --- |
| Family 1 Individual II:1 | 18 | Female | Prelingual | Profound | yes | Bilateral |
| Family 1 Individual II:2 | 16 | Male | Prelingual | Profound | yes | Bilateral |
| Family 1 Individual II:3 | 4 | Male | Congenital | Profound | No progression | Bilateral |
| Family 2 Individual II:1 | 28 | Female | Congenital | Profound | No information | Bilateral |
| Family 3 individual II:1 | 80 | Male | Congenital | Profound | No information | Bilateral |
| Family 3 individual II:3 | 74 | Male | Congenital | Severe | No information | Bilateral |
| Family 3 individual II:5 | 70 | Female | Congenital | Severe | No information | Bilateral |
| Family 3 individual II:7 | 68 | Female | Congenital | Severe | No information | Bilateral |
| Family 3 individual IV:1 | 13 | Male | Congenital | Severe | No information | Bilateral |
| Family 3 individual IV:3 | 19 | Female | Congenital | Severe | No information | Bilateral |
| Family 3 individual IV:5 | 17 | Male | Congenital | Severe | No information | Bilateral |
| Family 4 individual II:1 | 21 | Female | Prelingual | Severe to profound | yes | Bilateral |
| Family 4 individual II:2 | 15 | Female | Prelingual | Severe to profound | yes | Bilateral |

1. Age at last examination

**Table S2:** Neurologic evaluation of affected individuals

|  | **Family 1** | | | **Family 2** | **Family 3** | |
| --- | --- | --- | --- | --- | --- | --- |
| **Individual** | II:1 | II:2 | III:3 | II:1 | II:1 | II:3 |
| **Mental Status** | Alert, oriented | Alert, oriented | Alert | Alert, oriented | Alert, oriented | Alert, oriented |
| **Cranial Nerves** | Normal except for CN VIII –  Deafness | Normal except for CN VIII –  Deafness | Normal except for CN VIII –  Deafness | Normal except for CN VIII –  Deafness | Normal except for CN VIII –  Deafness | Normal except for CN VIII –  Deafness |
| **Motor Examination** | Normal | Normal | Normal | Normal | Normal | Normal |
| **Sensory examination** | Grossly normal | Grossly normal | Not done | Not done | Not done | Not done |
| **Coordination** | Normal | Normal | Normal | Normal | Normal | Normal |
| **Deep Tendon Reflexes** | Normal | Normal | Normal | Normal | Not done | Not done |
| Gait | Normal | Normal | Normal | Normal | Normal | Normal |
| Brain MRI | N/A | N/A | N/A | N/A | Normal | Normal |

**Table S3:** Genome and exome sequencing statistics

| **Family and**  **individual** | | **NGS performed** | **Platform** | **Average read depth** | **Coverage^2^** | **Coverage^3^** |
| --- | --- | --- | --- | --- | --- | --- |
| Family 1 | II:1 | Genome | BGI BGISEQ-500 | 47.32x | 99.20% | 98.88% |
|  | II:2 | Genome | Illumina X Ten | 36.19x | 99.60% | 99.60% |
|  | II:3 | Genome | BGI BGISEQ-500 | 35.17x | 99.60% | 99.20% |
| Family 2 | II:1 | Genome | Illumina NovaSEQ 6000 | 38.73x | 99.20% | 99.10% |
| Family 3 | IV:3 | Exome^1^ | Illumina HiSeq 2000 | 209.1x | 97.74% | 93.68% |
|  | IV:5 | Exome^1^ | Illumina HiSeq 2000 | 182.7x | 97.77% | 93.7% |
| Family 4 | II:1 | Exome^1^ | Illumina HiSeq 2000 | 216.7x | 97.84% | 93.91% |

1. Agilent SureSelect V7 capture
2. Coverage statistics: 1x for genome and Q20 for exome sequencing
3. Coverage statistics: 4x for genome and Q30 for exome sequencing

**Table S4:** Parameters used for detecting homozygous runs in whole genome sequencing data

| **Parameter** | **Genome sequencing** |
| --- | --- |
| Variation types to test | SNPs only |
| Ignore rare or common variations | <5% and >95% |
| Ignore variations that have a quality score less than | 20 |
| Consolidate nearby regions | Checked |
| Minimum region length | 50000 bp |
| Window scan size | 50 variations |
| Maximum number heterozygous variants in window scan | 1 |
| Percent of windows for homozygous position call | 5% |
| Number of consecutive positions to call a homozygous region | 100 |
| Maximum gap between variations in a region | 100000 bp |

**Table S5:** Shared homozygous runs >1 MB in three affected siblings of Family 1 (Genome Sequencing data)

| **Ind.** | **Region (Hg 19)** | **Length (bp)** | **1x coverage %** | **5x coverage %** | **avg read depth x** |
| --- | --- | --- | --- | --- | --- |
| **II:1** | chr5: 123,576,980-132,980,451 | 9,403,471 | 100 | 99.9 | 45.02 |
| **II:2** |  |  | 100 | 100 | 36.72 |
| **II:3** |  |  | 100 | 99.9 | 35.64 |

**Table S6:** 10 longest homozygous runs in the proband of Family 2 individual II:1 (Genome Sequencing data)

| **Shared homozygous region (hg19)** | **Length (bp)** | **1x coverage %** | **5x coverage %** | **avg read depth x** |
| --- | --- | --- | --- | --- |
| chr1: 69,420,375-83,464,725 | 14,044,351 | 99.6 | 99.6 | 38.55 |
| chr5: 128,253,080-141,730,596 | 13,477,517 | 99.5 | 99.5 | 38.18 |
| chr10: 64,139,582-73,149,445 | 9,009,864 | 100.0 | 99.9 | 38.25 |
| chr8: 118,676,625-126,694,576 | 8,017,952 | 100.0 | 100.0 | 38.56 |
| chr10: 82,594,703-90,233,592 | 7,638,890 | 100.0 | 99.9 | 38.62 |
| chr9: 214,706-6,553,054 | 6,338,349 | 100.0 | 100.0 | 38.66 |
| chr10: 74,262,292-79,466,258 | 5,203,967 | 100.0 | 100.0 | 38.31 |
| chr8: 46,870,230-52,072,125 | 5,201,896 | 99.8 | 99.8 | 38.86 |
| chr1: 22,348,587-26,579,223 | 4,230,637 | 99.6 | 99.2 | 37.16 |
| chr1: 33,482,763-37,403,014 | 3,920,252 | 100.0 | 100.0 | 38.20 |

**Table S7:** Characteristics of the identified *MINAR2* variants

|  | **Variant 1** | **Variant 2** | **Variant 3** |
| --- | --- | --- | --- |
| **Family ID** | Family 1 | Family 2 | Families 3 and 4 |
| **c. DNA position**  **(NM_001257308.2)** | c.412_419delCGGTTTTG | c.144G>A | c.393G>T |
| **a.a. notation**  **(NM_001257308.2)** | p.Arg138Valfs*10 | p.Trp48* | p.Lys131Asn |
| **Genomic location (hg19) (NC_000005.9)** | g.129100595_129100602delCGGTTTTG | g.129084027G>A | g.129096298G>T |
| **Variant type** | Frame-shift | Nonsense | Missense-Splice |
| **gnomAD global MAF** | absent | absent | absent |
| **dbSNP** | absent | absent | absent |
| **GERP RS score** | 3.608 (Mean) | 4.7199 | 4.7899 |
| **CADD Phred score** | NA | NA | 26.2 |
| **DANN score** | NA | 0.9952 | 0.9987 |
| **TraP Score** | NA | NA | 0.937 |

**gnomAD**: Genome aggregation database, **dbSNP**: The single nucleotide polymorphism database **GERP**: Genomic evolutionary rate profiling, **CADD:** Combined annotation dependent depletion, **DANN:** Deleterious annotation of genetic variants using neural networks, **TraP:** Transcript-inferred pathogenicity

**
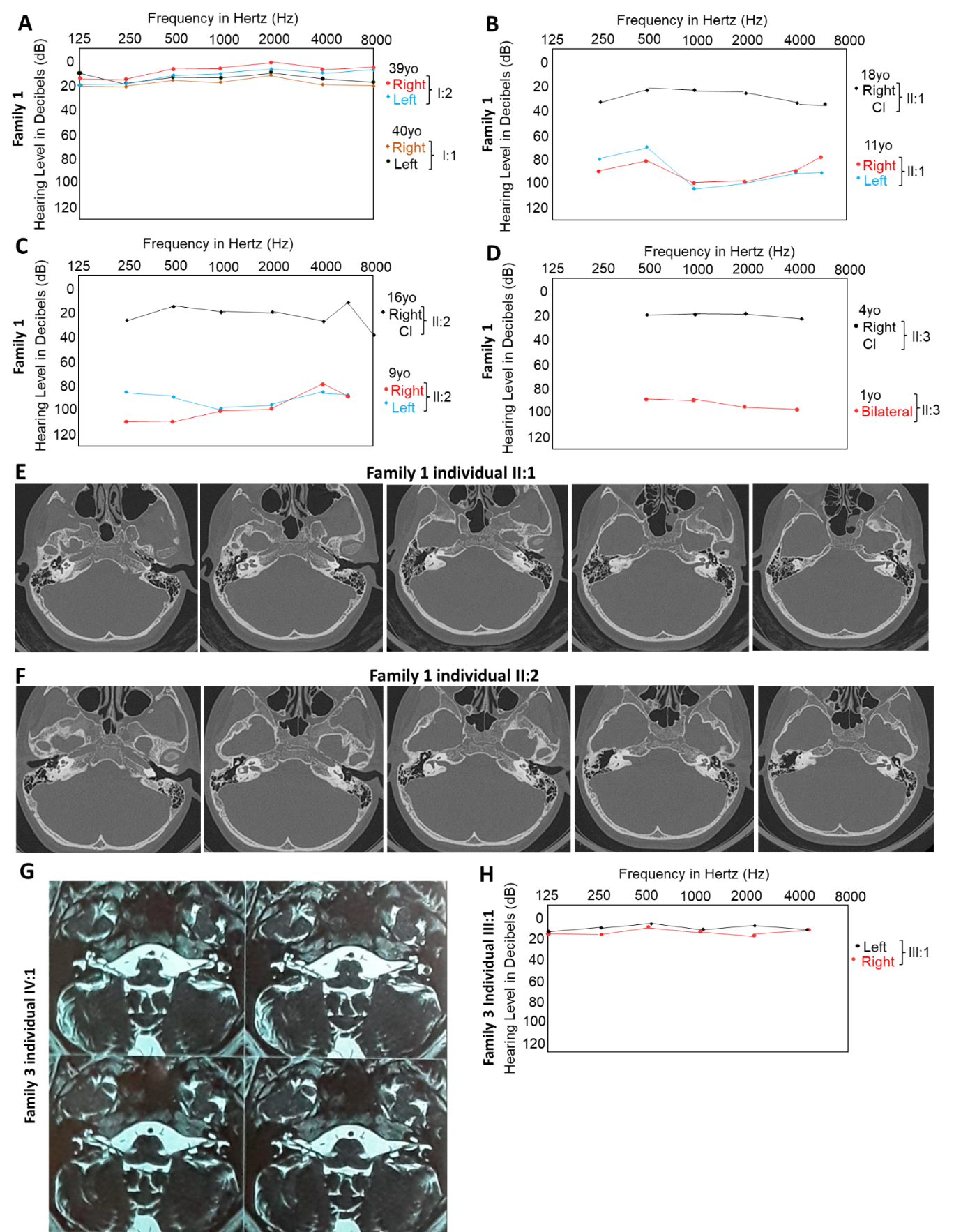
**

**Figure S1.** Audiogram and imaging features of families. **(A)** Audiograms of both parents which were heterozygous for *MINAR2* c.412_419delCGGTTTTG show normal hearing. **(B-D)** Audiograms before and after cochlear implants in three siblings of Family 1. **(E, F)** Temporal bone CT imaging of two members of Family 1 showing normal anatomic structures of the inner ear. **(G)** MRI imaging of the inner ear in an affected member of Family 3 showing normal structures. **(H)** Normal audiogram of a heterozygous parent (III:1) in Family 3. Please note that only reports of inner ear radiological studies (not images) are available for Families 2 and 4.

**
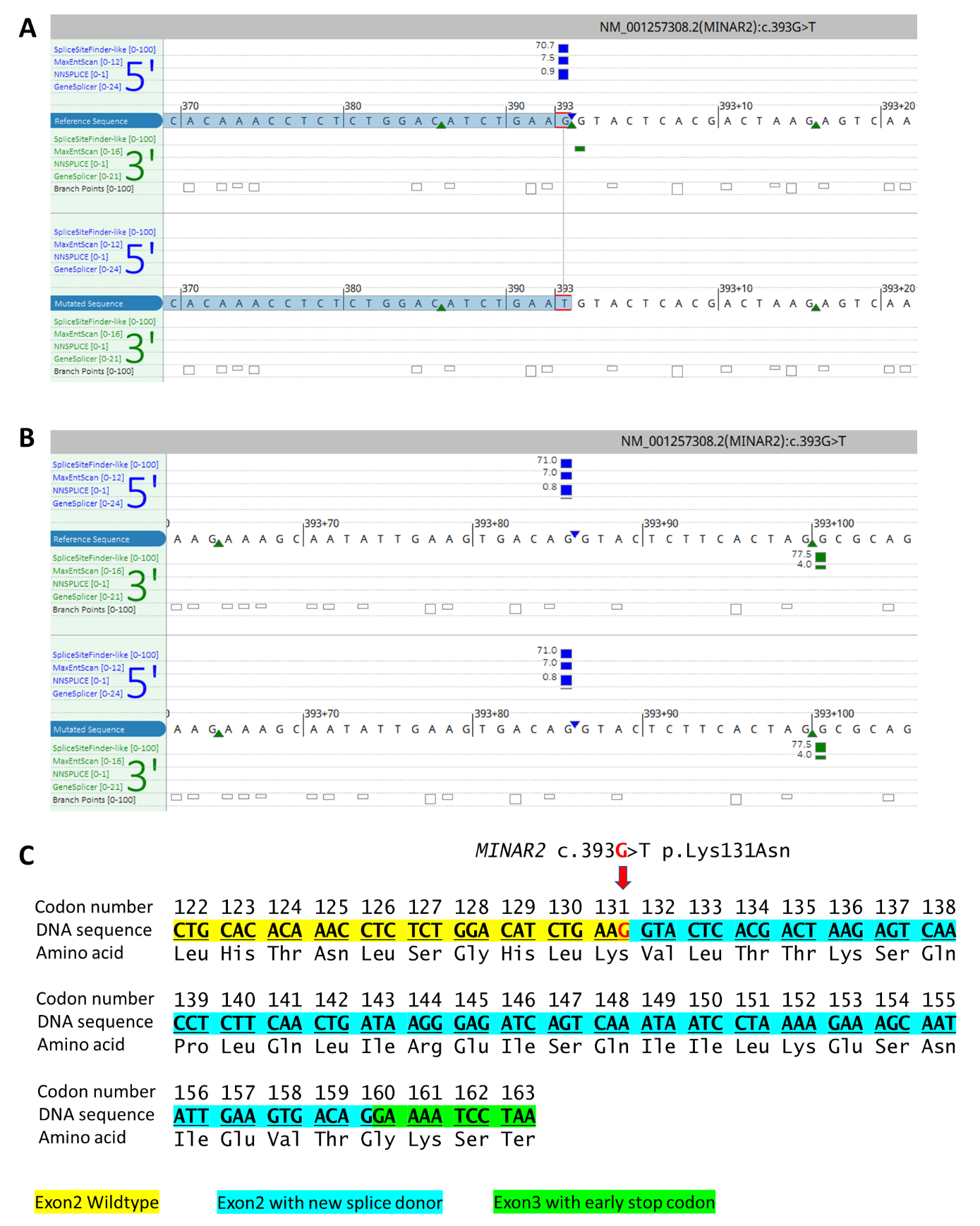
**

**Figure S2:** Effects of c.393G>T on splicing. **(A and B)** prediction tools SpliceSiteFinder-like, MaxEntScan and NNSPLICE show disruption of the wildtype donor and potential new donor sites. **(C)** Functional studies show that the variant c.393G>T shifts the donor site 85 bp into intron 2, utilizing a new donor splice site predicted in B (marked with blue) and causes early stop codon in exon 3.


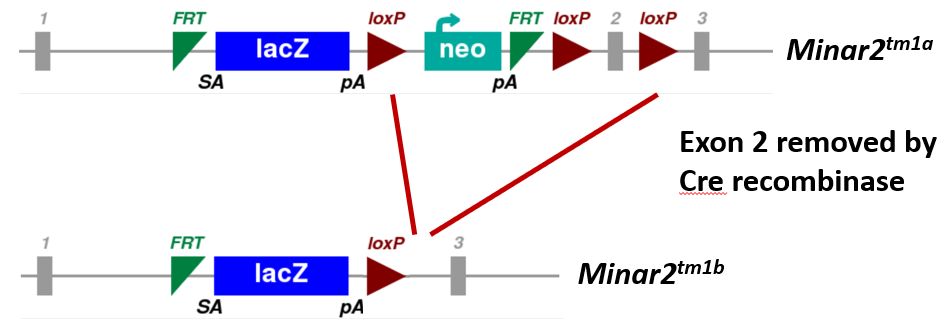


**Figure S3:** *Minar2* mutant mice. Schematic showing the conversion of the *Minar2^tm1a^* allele (top) to the *Minar2^tm1b^* allele (bottom) by exposure to Cre recombinase, which recombines between two LoxP sites (red triangles) deleting intervening DNA including exon 2 (grey box) of the gene. Green triangles, FRT sites. Blue box, LacZ gene. Turquoise box, neomycin selection marker. See <https://www.mousephenotype.org/data/genes/MGI:2442934> for further details.

**
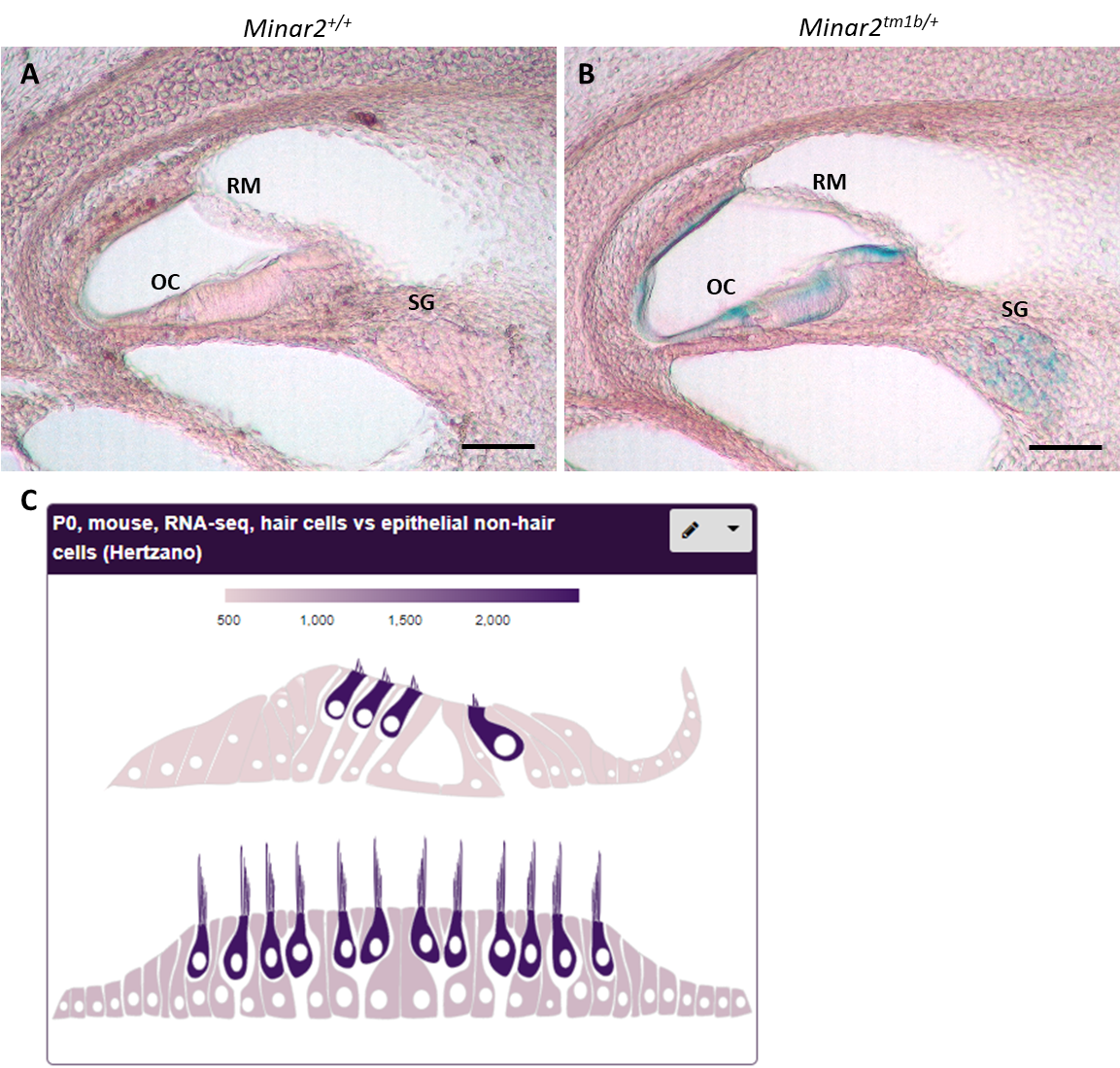
**

**Figure S4:** Expression pattern of *Minar2* using the reporter gene *LacZ* of the mutant allele and β-gal staining shown in blue. **(A)** Wildtype **(B)** Mutant. Note the localization of Minar2 at the SG: spiral ganglion, OC: organ of Corti, RM: Reissner’s membrane. Scale bar: 80 µm (Both images) **(C)** Relative expression pattern of *Minar2* (previously known as A730017C20Rik) in the mouse inner ear (data from Elkon et. al., 2015) in the gene Expression Analysis Resource (gEAR; <https://umgear.org/>).


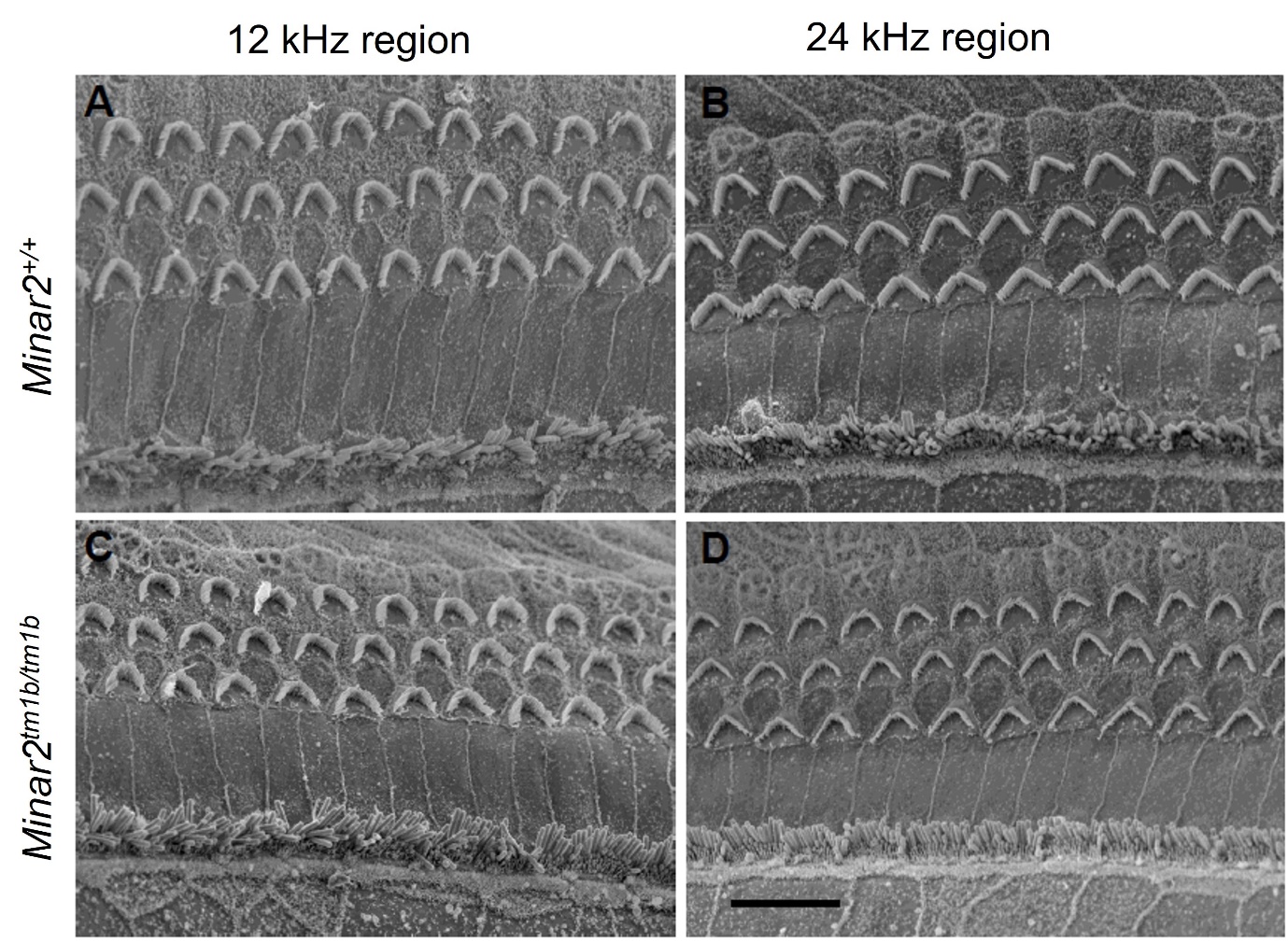


**Figure S5:** Scanning electron microscopy showing normal arrangement of cells within the organ of Corti. **(A, B)** Wildtype organ of Corti at best frequency locations for 12 kHz (70% of distance from base) and 24 kHz (40% distance from base). **(C, D)** *Minar2^tm1b^* homozygotes at 12 kHz and 24 kHz best frequency locations, showing similar arrangement of cell types. Scale bar (in **D**) indicates 10μm.


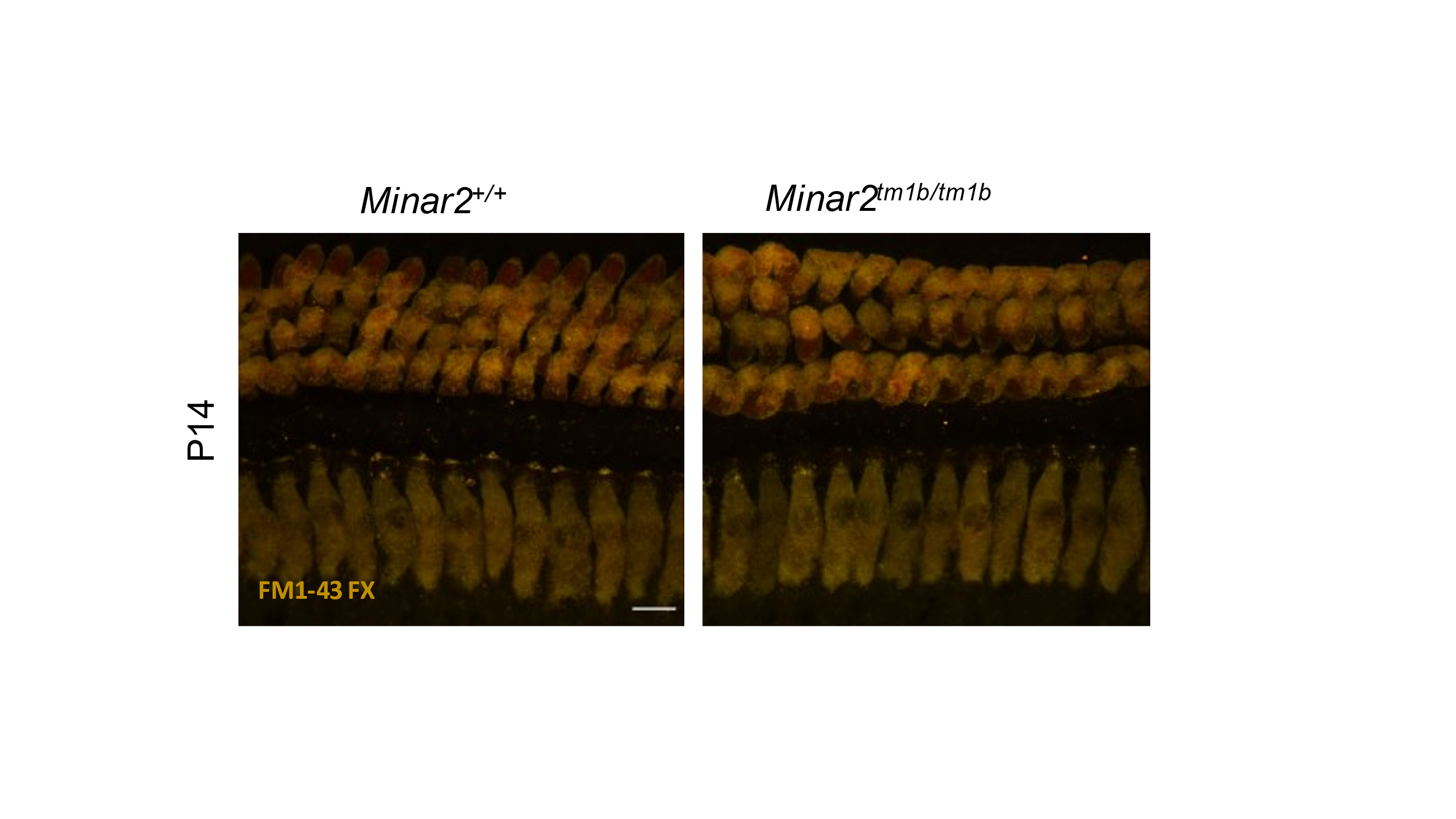


**Figure S6:** FM1-43 FX uptake in *Minar2* mutant mice. Representative confocal images of the FM1-43 FX uptake in hair cells of *Minar2^+/+^* and *Minar2^tm1b/tm1b^* mice (n = 4 for each genotype) at P14. The images correspond to the 24 kHz region. The frequency regions of 12 and 36 kHz were also analyzed, but no differences were found between different frequencies. The FM1-43 dye emits fluorescence at wavelengths corresponding to red and green. Here, the merge of both channels is shown. Scale bar = 10 µm.

**
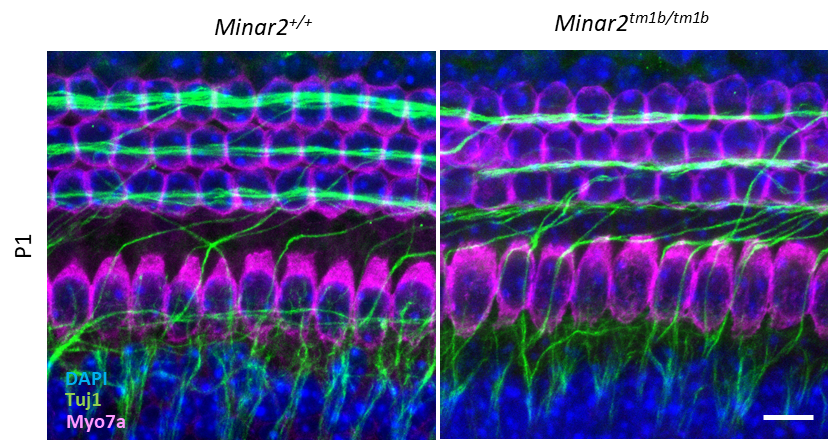
**

**Figure S7:**   Confocal maximum intensity projection images from 24 kHz region of the whole-mount organ of Corti. Total innervation with anti-Neurofilament label (green) and Myo7a (magenta) is shown for P1 (n=2 each group) wild type and mutant mice; bar=10µm.

**
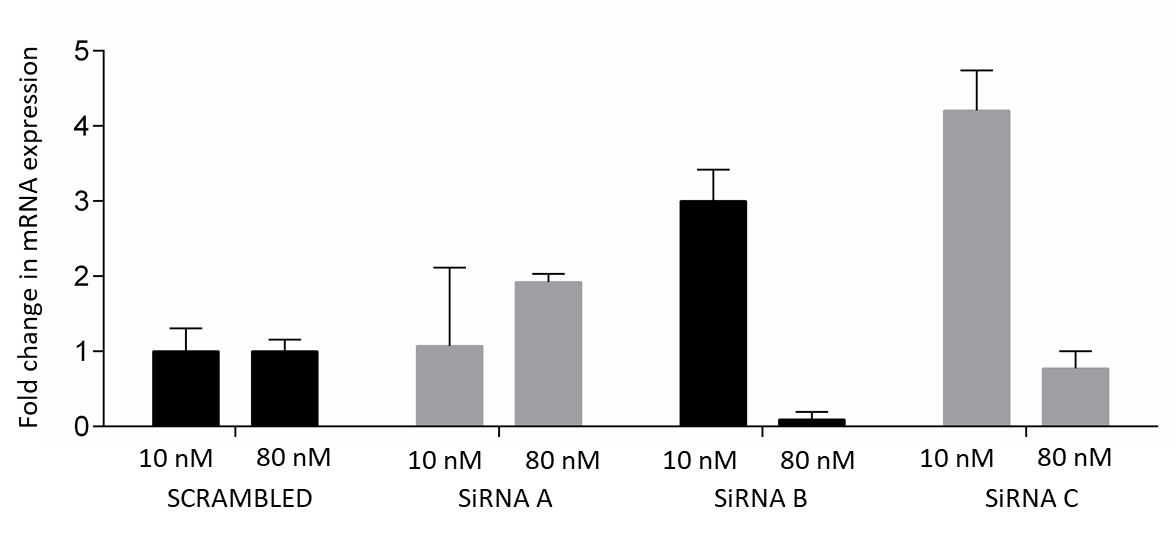
**

**Figure S8:** qRT-PCR analysis of effect of scrambled and three predicted *MINAR2* SiRNA sequences namely A, B, and C at concentrations of 10 nM and 80 nM on *MINAR2* mRNA expression in PC12 cells after 48 hrs of transfection, respectively. The results are expressed as fold change in mRNA levels±SEM.


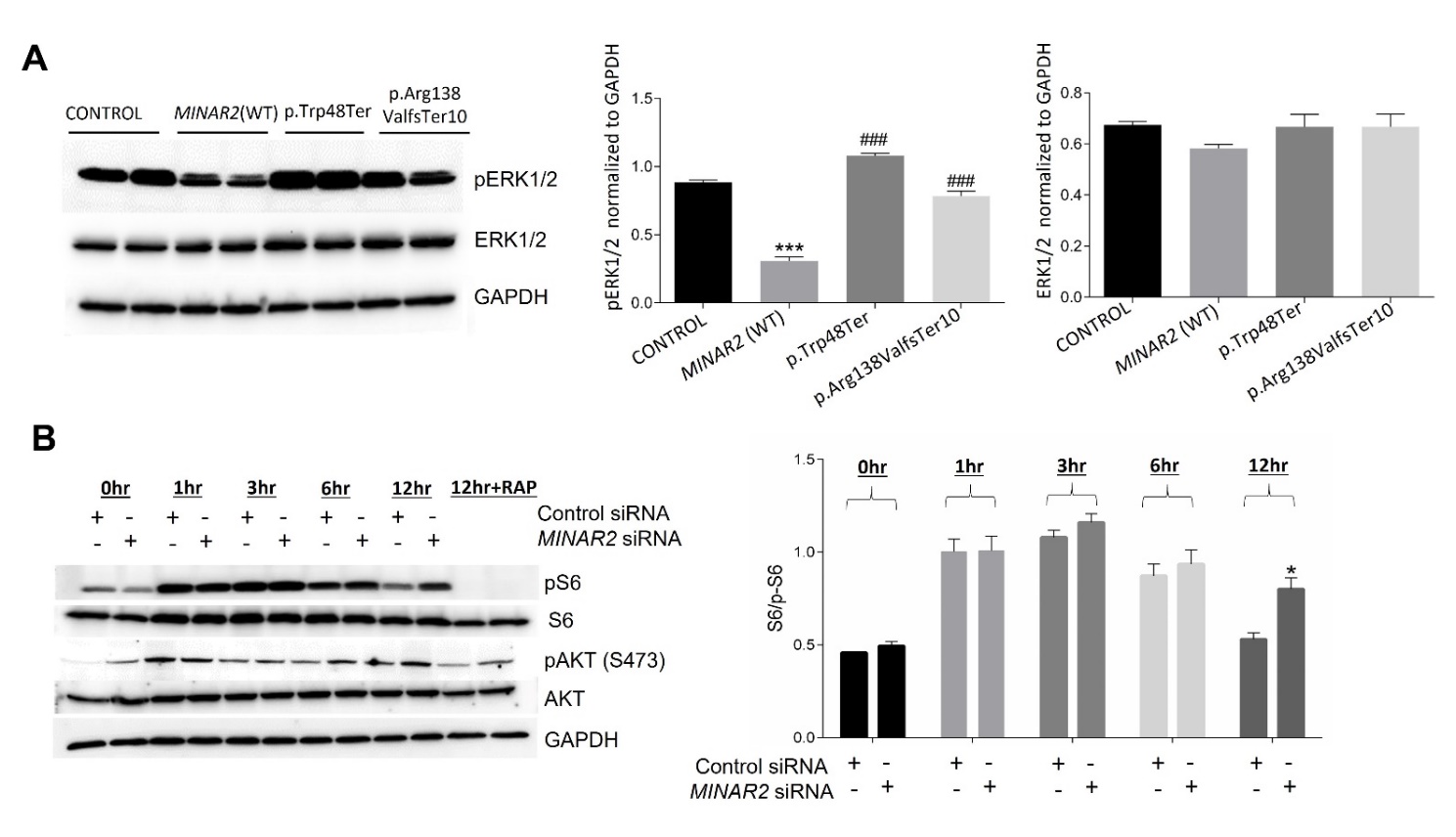


**Figure S9.** Effects of *MINAR2* on ERK, S6, and AKT. **(A)** Left: western blot images of *MINAR2* (WT), p.Trp48*, and p.Arg138Valfs*10 variants transient overexpression on phosphorylation of Extracellular signal-regulated protein kinases 1 and 2 (ERK1/2); Right: statistical analysis of GAPDH-normalized relative protein expressions of pERK1/2 and ERK1/2. Results are expressed as mean±SEM and significant differences are shown as *** (p≤0.001) when compared to control and as ### (p ≤0.001) when compared to MINAR*2(WT).* **(B)** PC12 cells transfected with either scrambled negative control siRNA or MINAR2 siRNA. Left: immunoblot analysis of S6 Ribosomal Protein and Akt along with their phosphorylation at Ser235/236, and Ser 473, respectively. Transfected cells were serum starved and treated with 50 ng/ml of NGF for 0 to 12 hours. Cell lysates were harvested after 48 hrs of siRNA incubation and at 0,1,3 and 12 hours of NGF treatment. Cells were treated with 100 nM of rapamycin (Rap) for 30 min after 12 hours of NGF treatment. GAPDH was used as a loading control. Right: quantitative analysis of S6/pS6 ratios in PC12 cells transfected with scrambled and MINAR2 siRNA at 0,1,3, and 12 hours of NGF treatment from three biological repeats. Results are expressed as mean±SEM and significant difference between scrambled siRNA and MINAR2 siRNA are shown as * (p≤0.05) at different time points. Data analyzed by one way ANOVA with multiple comparisons.

**Supplemental Materials and Methods**

**Human Subjects.**

The diagnosis of sensorineural HL was established via auditory brainstem response or standard audiometry in a sound-proof room according to the current clinical standards. Clinical evaluation included reviews of past medical history with an emphasis on environmental causes of HL and syndromic deafness and a thorough physical examination including an eye exam and otoscopy in all cases. DNA was extracted from peripheral blood leukocytes of participants according to the standard procedures.

**DNA Sequencing and Bioinformatics Analysis.**

Combined Annotation Dependent Depletion (CADD), deleterious annotation of genetic variants using neural networks (DANN), Transcript-inferred Pathogenicity (TraP) and Genomic Evolutionary Rate Profiling (GERP) scores used for *in silico* prediction of the identified variants (1-4). NNSplice, MaxEntScan, and Splice site Finder-Like used through Alamut Visual Plus version 1.1 software (https://www.interactive-biosoftware.com/alamut-visual-plus/) for the further splicing prediction (5-7).

**Site directed mutagenesis.**

*MINAR2* (OHu01804, NM_001257308.2) cDNA cloned in pcDNA3.1+/C-(K)DYK was procured from Genscript Biotech (NJ, USA). Site-directed mutagenic changes to incorporate c.412 _ 419delCGGTTTTG and c.144G>A were made using Quick Change Lightning Multi Site-Directed Mutagenesis Kit (Agilent Technologies, USA). Briefly, 40 ng plasmid was subjected to PCR amplification as per standard kit guidelines using mutagenic primers 5'-agtgtacatgtctcccaaccaggtcattaggattttcc-3', 5'-ggaaaatcctaatgacctggttgggagacatgtacact-3', and 5'-gagtagacaaggttttgtcagtgttgtgcagcagg-3', 5'-cctgctgcacaacactgacaaaaccttgtctactc-3' for c.412_419delCGGTTTTG and c.144G>A variants, respectively. After PCR, 10ul of the product was digested by DpnI for 5 min at 37 ^o^C and transformed into chemically competent DH5-alpha cells (NEB, USA) by heat shock at 42 ^o^C for 30 sec. Resulting transformants were grown in SOC media for 1hr at 37 ^o^C and selected overnight on LB agar plates containing 100 µg/ml Ampicillin. Following day, single colonies were selected and further grown in LB broth containing 100 µg/ml Ampicillin for 12 hours. Plasmid isolation and purification were carried out in accordance with standard instructions using plasmid mini-prep kit (Qiagen, Germany). Sanger sequencing for confirmation of variant incorporation was performed in Genewiz, USA; by using cytomegalovirus virus forward primer (5'-CGCAAATGGGGTAGGCGTG-3').

**Minigene assay for *MINAR2* c.393G>T and transfections in HEK293.**

The preparation of minigene was carried out with the pET01 vector (Mobitec, GmbH). This Exontrap system is based on a shuttle vector (pET01), which already contains 5’ and 3’ exon separated by a 600 bp intron sequence, including a multiple cloning site (MCS). To include MINAR2 c.393G>T, 528 bp regions including exon2 and flanking introns were amplified using primers 5’-CCGCTCGAGACGAAACCCTAGAATCCTTTT-3’ and 5'- TCCCCGCGGGAGAAAGAAAAGCCAG-3', which contain overhangs for XhoI and SacII restriction sites. After amplification, bands were excised, digested, and ligated with T4 DNA ligase into pET01. After ligation, the resulting recombinant pET01 was transformed and selected on ampicillin plates 100 µg / ml.

Positive colonies were identified, and isolated-recombinant vectors were confirmed using Sanger sequencing primer 5'-GCGAAGTGGAGGATCCACAAG-3. Furthermore, HEK-293 cells were transfected with 2 µg of empty vector pET01, pET01 containing control fragment, and Mutant *MINAR2* c.393G>T fragments. RNA isolation was done 48 hours after the initial transfection, and reverse transcription was done using SuperScript™ IV First-Strand Synthesis System (Thermo Fisher, USA) and cDNA primer 5’-GATCCACGATGC-3' specific to pET01. RT-PCR was then performed with 5 'GATGGATCCGCTTCCTGCCCC-3,' and 5 'CTCCCGCCACCTCAGTGCC-3', and the resulting product was analyzed using agarose (1.3%) gel electrophoresis. Confirmatory Sanger sequencing performed with 5 'GATGGATCCGCTTCCTGCCCC-3,' and 5 'CTCCCGCCACCTCAGTGCC-3' primers and analyzed with Sequencher 5.4.6 (<http://www.genecodes.com/>) software.

**Genotyping in Mice.**

Mice were genotyped by PCR using template DNA extracted from pinna skin. The mutant allele was amplified using a gene-specific forward primer (5’-CAATCCCCACTCCCAAACTC-3’) paired with the reverse primer CasR1 (5’- TCGTGGTATCGTTATGCGCC-3’). The wildtype allele was genotyped in a similar way using the same forward primer combined with a reverse primer specific for the wildtype allele (5’-ACAACCCCTTGATCTGCCTC-3’). The *Minar2^tm1b(KOMP)Wtsi^* allele was detected by a primer pair recognizing the LacZ gene (primer F: 5’-ATCACGACGCGCTGTATC-3’, primer R: 5’-ACATCGGGCAAATAATATCG-3’). An extra primer set to amplify exon 2 was designed to distinguish the wildtype and mutant alleles (primer F: 5’- AGAGTCCTCCAGCATCCATG-3’, primer R: 5’- tgctttctaggttccggtca-3’).

***Minar2* mRNA expression in Mice.**

To check the expression of *Minar2* in different tissues, lung, liver, kidney, brain and cochlea were dissected from P30 wildtype mice. In addition, the cochlear expression of *Minar2* was assessed in E18.5 and P0 samples. Total RNA was isolated with TRIzol Reagent (Invitrogen) according to manufacturer’s instructions. Prior to reverse transcription, RNA samples were treated with rDNAse I (DNA-free kit, Applied Biosystems). cDNA was synthetized using qScript XLT cDNA SuperMix (Quanta Biosciences). The primers used to amplify a 117 bp fragment of the *Minar2* transcript were: forward 5′-TGGACCATTGAGGAGTATGACA-3′ and reverse 5′-GTCGAAGCCAGGAGTGTACG-3’. For *Gapdh*, a 171 bp fragment was amplified with 5’-ACCCAGAAGACTGTGGATGG-3’ forward primer and 5’-CACATTGGGGGTAGGAACAC-3’ reverse primer.

**Auditory Brainstem Response (ABR) measurements in Mice.**

ABRs were recorded as described previously (8, 9). Mice were anesthetized using intraperitoneal 100 mg/kg Ketamine (Ketaset, Fort Dodge Animal Health) and 10 mg/kg Xylazine (Rompun, Bayer Animal Health), and sub-cutaneous recording needle electrodes (NeuroDart; UNIMED UK) were inserted on the vertex and overlying the left and right bullae. Free-field click stimuli (10 µs duration) and tone pips (5 ms duration, 1ms onset/offset ramps) at frequencies from 3 to 42 kHz and sound levels from 0-95 dB SPL in 5 dB increments were presented at 42.6 stimuli per second. Evoked responses were amplified, digitized, and bandpass filtered between 300-3000 Hz. Thresholds were defined as the lowest stimulus level which evoked a response waveform with characteristics features, determined by visual inspection. Homozygous mutant mice and littermate wildtype controls were tested at post-natal day (P) 14, P21 and P27-28.

**Distortion Product OtoAcoustic Emission (DPOAE) measurements in Mice.**

Following ABR measurements DPOAEs were measured. The microphone probe tip of an Etymotic ER10B+ low noise system was positioned next to the opening of the left ear canal. Using Tucker Davis Technologies BioSigRZ software and MF1 loudspeakers, f2 tones were presented at 6, 12, 18, 24 and 30 kHz at increasing level, -10 to 75 dB SPL in 5 dB steps, whilst f1 tones were presented at 10 dB above the f2 (0 to 85 dB SPL), at a frequency ratio of 1 : 1.2 for f1 : f2 tones. For each f2 frequency the DPOAE threshold was determined at the lowest f2 stimulus level (dB SPL) where the 2f1-f2 DPOAE rose in amplitude above two standard deviations from the mean noise floor.

**Endocochlear Potential (EP) recording in Mice.**

EP was measured in urethane-anaesthetised mice (0.1 ml/10g bodyweight of a 20%w/v solution) aged P30-32 and 14 weeks old using 150 mM KCl-filled glass pipette microelectrodes, as described previously (10, 11). EP was recorded as the potential difference (mV) between the tip of a glass microelectrode inserted into scala media via a fenestration in the cochlea basal turn lateral wall and a reference Ag-AgCl pellet electrode inserted under the skin of the dorsal surface of the neck.

**Wholemount dissection, immunohistochemistry, and confocal imaging of synapses in Mice.**

Cochleae of postnatal day (P)14, P30-32 and 14-week old mice were fixed in 4% paraformaldehyde (PFA) in PBS for 2h and decalcified with 0.1M ethylenediaminetetraacetic acid (EDTA) overnight at room temperature (RT). After dissection of the organ of Corti, P14 and P30-32 samples were permeabilized with 5% Tween in PBS for 30 min and blocked in 0.5% Triton X-100 and 10% Normal Horse Serum (NHS) in PBS for 2h. The 14-week old samples were blocked in 10% horse serum diluted in 0.3% Triton X-100. Then, the tissues were incubated overnight with the primary antibodies diluted in 8% NHS (5% for the 14-week old samples) at 4^o^C . The primary antibodies used were rabbit anti-Myosin VIIa (diluted 1:200; 25-6790, Proteus), mouse IgG2 anti-glutamate receptor 2 (GluR2) (diluted 1:2000; MAB397, Emd Millipore) and mouse IgG1 anti-C-terminal-binding protein 2 (CtBP2) (diluted 1:200; 612044, BD Transduction Laboratories). Samples were incubated with the secondary antibodies for two cycles of 1h at 37^o^C. The secondary antibodies used were Alexa Fluor 647-conjugated chicken anti-rabbit (1:200; #A21443, Thermo Fisher Scientific), Alexa Fluor 488-conjugated goat anti-mouse (IgG2a) (diluted 1:1000; #A21131, Thermo Fisher Scientific) and Alexa Fluor 568-conjugated goat anti-mouse (IgG1) (1:1000; #A21124, Thermo Fisher Scientific). For the 14-week old samples, rhodamine phalloidin (1:500, #R415, Thermo Fisher Scientific) was used in combination with Alexa Fluor 488-conjugated goat anti-rabbit (1:300, #A11008, Thermo Fisher Scientific) for 1h at RT. Specimens were mounted using ProLong Gold Antifade Mountant with DAPI (P36931, Life Technologies) and stored at 4^o^C.

For synaptic studies, samples were imaged with a Zeiss LSM 700 inverted confocal microscope interfaced with ZEN 2011 software (v14.0.17.201). All the images were captured with the plan-Apochromat 63x/1.40 oil DIC objective and 2.0 optical zoom. Confocal z-stacks were obtained with a z-step size of 0.25 μm, ensuring that all the synaptic puncta were imaged. Images were acquired at the 12 kHz and 24 kHz best-frequency regions and the ImageJ measure line plug-in (Eaton Peabody Laboratories) was used to map cochlear length to cochlear best frequencies. Brightness and contrast levels were adjusted for whole images using Fiji software. Synaptic puncta counts were performed manually using the cell-counter plugin in Fiji software. All the images containing puncta were merged in a z-stack and the z-axis maximum intensity projection was used for quantification of synapses, defined by colocalization of CtBP2- and GluR2-labelled puncta. The total number of ribbon synapses was divided by the number of Myo7a-labelled IHCs present in the image to determine the number of ribbon synapses per IHC. In the cases where an IHC was not completely visible in the image, the synapses corresponding to that cell were not counted. After counting of synapses, whole images were subjected to the smoothing function of Fiji for presentation. Two non-overlapping images were acquired at each best-frequency region, counting between 10 to 14 IHCs at each frequency. A one-way ANOVA with multiple comparisons was used to compare the different experimental groups. Images of 14-week old samples were acquired with a Zeiss Imager 710 confocal microscope interfaced with ZEN 2010 software. First, overview images of all samples were captured with the x20 objective, using the tile function to capture whole pieces in single images. Then, the tiles were stitched during post-processing, using a medium correlation threshold (0.7) so that all the tiles were converted into individual images. Higher magnification images of specific regions of interest were acquired using the plan-Apochromat 63x Oil DIC objective, and the z-stack interval in these cases was 1 µm.

**Additional immunofluorescence in Mice.**

To perform additional experiments, wildtype mice were culled by decapitation (P1) or by cervical dislocation (P14 and P30). The skull was cut in two halves and the brain was removed to facilitate the penetration of the fixative. Samples were washed with cold PBS, fixed overnight in 4% PFA at 4^o^C and then washed twice with cold PBS. Tympanic bullae containing the cochleae were dissected from P1 mice under the microscope and locally perfused with 4% PFA through the round and oval windows. Samples were kept in 4% PFA at 4 °C overnight and rinsed in 1X PBS. Cochlea whole mounts were permeabilized with 0.5% Triton X-100 and blocked in 5% BSA for 1 hour at room temperature, followed by overnight incubation at 4°C with primary antibodies. A mouse anti-Tuj1 (MMS-435P) and a chicken anti-Beta Galactosidase (ab9361) were utilized as a primary antibody for *Minar2^+/tm1b^* mice. A mouse anti-Myo7a (MYO7A 138-1, DSHB), a chicken anti-NF (AB5539, Millipore), a rat anti-Notch2 (MA5-24274, Invitrogen), and a mouse anti-2H3 (2H3-s, DSHB) were utilized as a primary antibody for *Minar2^+/+^* and *Minar2^tmb1b/tm1b^* mice. Specimens were washed with PBS, and then incubated with anti-mouse Alexa Fluor 568 and 488, and anti-chicken 647 were applied. DAPI (Calbiochem) were used to counterstain a nuclear DNA. Specimens were washed with PBS and mounted in fluorescence mounting medium (Dako). Images were taken using a Zeiss LSM 710 fluorescence microscope. ImageJ software (National Institute of Health) was used to quantify the fluorescence intensity and to count the number of type II crossings.

**X-gal staining in Mice.**

Whole mount cochlea aged P1 were incubated overnight at 37°C in X-gal staining solution (0.02% NP40, 0.01% Sodium Deoxycholate, 2mM MgCl_2_, 5mM K_3_Fe(CN)_6_, 5mM K_4_Fe(CN)_6_, 0.5 mg/ml X-gal), washed twice with PBS 1-X and mounted in mounting medium (Dako).

**Scanning electron microscopy.**

Two wildtype, two heterozygous and three homozygous mutant mice at P14 were used. The inner ears were fixed in 2.5% glutaraldehyde in 0.1M sodium cacodylate buffer with 3 mM calcium chloride at room temperature for 2-3 hours. Cochleae were further dissected in PBS to expose the surface of the organ of Corti, followed by processing using an osmium-thiocarbohydrazide-osmium (OTOTO) method (12). The samples were dehydrated in increasing concentrations of ethanol, critical-point dried (Leica-microsystems EM CPD300) and mounted. After sputter coating with 4nm gold (Leica EM ACE600), the samples were examined under a JEOL JSM 7800F Prime scanning electron microscope at the King’s College London Centre for Ultrastructural Imaging. Each sample was examined along the entire length of the cochlear duct and images were collected to correspond to specified best-frequency locations (13).

**FM1-43 dye uptake experiments.**

Cochleae from P14 *Minar2^tm1b/tm1b^* mice and littermate controls were dissected in Leibovitz’s L-15 medium (#11415064, Thermo Fisher Scientific). Each cochlea was perfused with 0.5 ml of FM1-43FX dye (fixable analog of FM™ 1-43 membrane stain, #F35355, Thermo Fisher Scientific) at 5 μM through incisions made in the round window, the oval window and the apex and incubated in the dye for 90 s. After incubation, tissue was washed 5 times with L-15 media, fixed in 4% paraformaldehyde (PFA) for 2 h at room temperature and decalcified with 0.1M EDTA overnight at RT. Following fine dissection, samples were mounted and imaged on a Zeiss LSM 700 inverted confocal microscope interfaced with ZEN2011 software. To image these samples, we used a 63x/1.40 oil DIC objective and a z-step of 1 μm. The frequency regions corresponding to 12, 24 and 36 kHz were imaged, and identical image acquisition settings were used to allow comparison between samples.

**In vitro angiogenesis assay of HUVEC cells.**

HUVEC cells were cultured in gelatin coated flasks using the EndoGRO endothelial cell growth kit (SCME002, Sigma-Aldrich). For transient transfections with plasmid constructs, 3x10^5^ cells were plated on 1% gelatin coated 6 wells plates. After 12 hours of initial plating, HUVEC cells were transiently transfected with 2 µg of *MINAR2* wildtype or *MINAR2* mutant constructs (c.412_419delCGGTTTTG and c.144G>A) using Jetprime transfection reagent (Polyplus-transfection® SA).

For comparative analysis of angiogenic potential of transiently transfected HUVEC cells with *MINAR2* constructs, 7x10^3^ cells were plated on Matrigel coated 6 well plates, as per user instructions of In vitro Angiogenesis Assay Kit (ECM625, Millipore Sigma). Plates were incubated at 37^o^C in humidified CO_2_ incubator for 12hrs. Images were acquired at 200µm with Leica DMIL inverted microscope, and analysis of the total length of angiogenic vessels was performed with Angiogenesis Analyzer for ImageJ, as described by Carpentier et al. (14).

**Cell culture and SiRNA induced silencing in PC12 cells.**

PC12 cells were grown in poly-D-lysine coated T-75 tissue culture flasks at 37^o^C in 5% CO2 using RPMI-1640, supplemented with 5% horse serum, 5% fetal bovine serum, and 1X penicillin and streptomycin. Cells were fed 3 times a week and were passaged at 80%-85% confluency. After 3 passages, 2.7x10^5^ cells were plated per well on poly-D-lysine coated 6 well plates and allowed to attach for 6-8 hrs. Cells were then washed using 1X Dulbecco’s PBS and incubated with antibiotics free fresh growth media containing 80 nM *MINAR2* siRNA (GUCAUGCUGGUGGAUUGAUAGAAAC) complexed with 7.5µl RNAiMAX transfection reagent (ThermoFisher, USA) for 24hrs. After 48 hours of transfection, cells were subjected to serum starvation in RPMI containing 1% horse serum, 50 ng/ml NGF (2.5S, Gibco Thermo Scientific, USA) and 1X antibiotics. To check the efficacy of *MINAR2* SiRNA in silencing *MINAR2* mRNA levels, cells were harvested in buffer RLT after 48 hours of initial transfection, and mRNA isolation was done as per user instructions of RNeasy Micro kit (Qiagen, Germany). cDNA was synthesized using qScript XLT cDNA SuperMix (Quanta Biosciences). The qRT-PCR reaction and cycle durations were set up as per user instructions of PowerUp SYBR Green Master mix using primer 5’-ATGCTGCCCTTTAGAC-3’; 5’GACAGACCCAGACTGGAATAAC-3’ for *MINAR2* and 5’ACTCCCATTCTTCCACCTTTG-3’; 5’-CCCTGTTGCTGTAGCCATATT-3’ for *GAPDH*. Efficiency of silencing is shown in ***SI Appendix*, Fig. S8.**

**Western blotting.**

Cells were harvested in RIPA buffer containing 1X protease and phosphatase inhibitor (Halt, Thermo Scientific, USA). Protein estimation of samples was done using commercially available BCA kit (Pierce, Thermo Scientific, USA). Equal concentration of protein was reduced and loaded on 4-20% Tris-Glycine gradient gel, and protein separation was done as per the method of Laemmli et al. (15). Following separation, protein was transferred onto 0.22 micron membrane using the Turbo-trans Blot system (Biorad, USA). The resulting PVDF membranes were blocked in 5% BSA for 1.5hr and incubated overnight at 4 °C in primary antibodies diluted (1:1000) in TBST (TBS+Tween 0.5%). Blots were washed with TBST and incubated in HRP conjugated Anti-Rabbit Goat secondary antibody (1:3000) for 1.5hr at room temperature. On termination of antibody reactions, blots were washed three times with TBST and developed and visualized using west pico super-signal ECL substrate (Pierce, Thermo, USA) and FluorChemE (ProteinSimple, USA) respectively.

**Statistics.**

A one-way ANOVA with multiple comparisons was used to compare groups for count of synapses in mouse inner ear and was also used for angiogenesis, MAPK, and mTOR assays.

**Supporting References**

1. G. M. Cooper *et al.*, Distribution and intensity of constraint in mammalian genomic sequence. *Genome Res* **15**, 901-913 (2005).

2. S. Gelfman *et al.*, Annotating pathogenic non-coding variants in genic regions. *Nat Commun* **8**, 236 (2017).

3. D. Quang, Y. Chen, X. Xie, DANN: a deep learning approach for annotating the pathogenicity of genetic variants. *Bioinformatics* **31**, 761-763 (2015).

4. P. Rentzsch, D. Witten, G. M. Cooper, J. Shendure, M. Kircher, CADD: predicting the deleteriousness of variants throughout the human genome. *Nucleic Acids Res* **47**, D886-D894 (2019).

5. M. G. Reese, F. H. Eeckman, D. Kulp, D. Haussler, Improved splice site detection in Genie. *J Comput Biol* **4**, 311-323 (1997).

6. M. B. Shapiro, P. Senapathy, RNA splice junctions of different classes of eukaryotes: sequence statistics and functional implications in gene expression. *Nucleic Acids Res* **15**, 7155-7174 (1987).

7. G. Yeo, C. B. Burge, Maximum entropy modeling of short sequence motifs with applications to RNA splicing signals. *J Comput Biol* **11**, 377-394 (2004).

8. N. J. Ingham, Evoked Potential Recordings of Auditory Brainstem Activity in the Mouse: An Optimized Method for the Assessment of Hearing Function of Mice. *Bio Protoc* **9**, e3447 (2019).

9. N. J. Ingham, S. Pearson, K. P. Steel, Using the Auditory Brainstem Response (ABR) to Determine Sensitivity of Hearing in Mutant Mice. *Curr Protoc Mouse Biol* **1**, 279-287 (2011).

10. N. J. Ingham *et al.*, S1PR2 variants associated with auditory function in humans and endocochlear potential decline in mouse. *Sci Rep* **6**, 28964 (2016).

11. K. P. Steel, C. Barkway, Another role for melanocytes: their importance for normal stria vascularis development in the mammalian inner ear. *Development* **107**, 453-463 (1989).

12. I. M. Hunter-Duvar, A technique for preparation of cochlear specimens for assessment with the scanning electron microscope. *Acta Otolaryngol Suppl* **351**, 3-23 (1978).

13. M. Muller, K. von Hunerbein, S. Hoidis, J. W. Smolders, A physiological place-frequency map of the cochlea in the CBA/J mouse. *Hear Res* **202**, 63-73 (2005).

14. G. Carpentier *et al.*, Angiogenesis Analyzer for ImageJ - A comparative morphometric analysis of "Endothelial Tube Formation Assay" and "Fibrin Bead Assay". *Sci Rep* **10**, 11568 (2020).

15. U. K. Laemmli, Cleavage of structural proteins during the assembly of the head of bacteriophage T4. *Nature* **227**, 680-685 (1970).

Uncropped Gel images

**
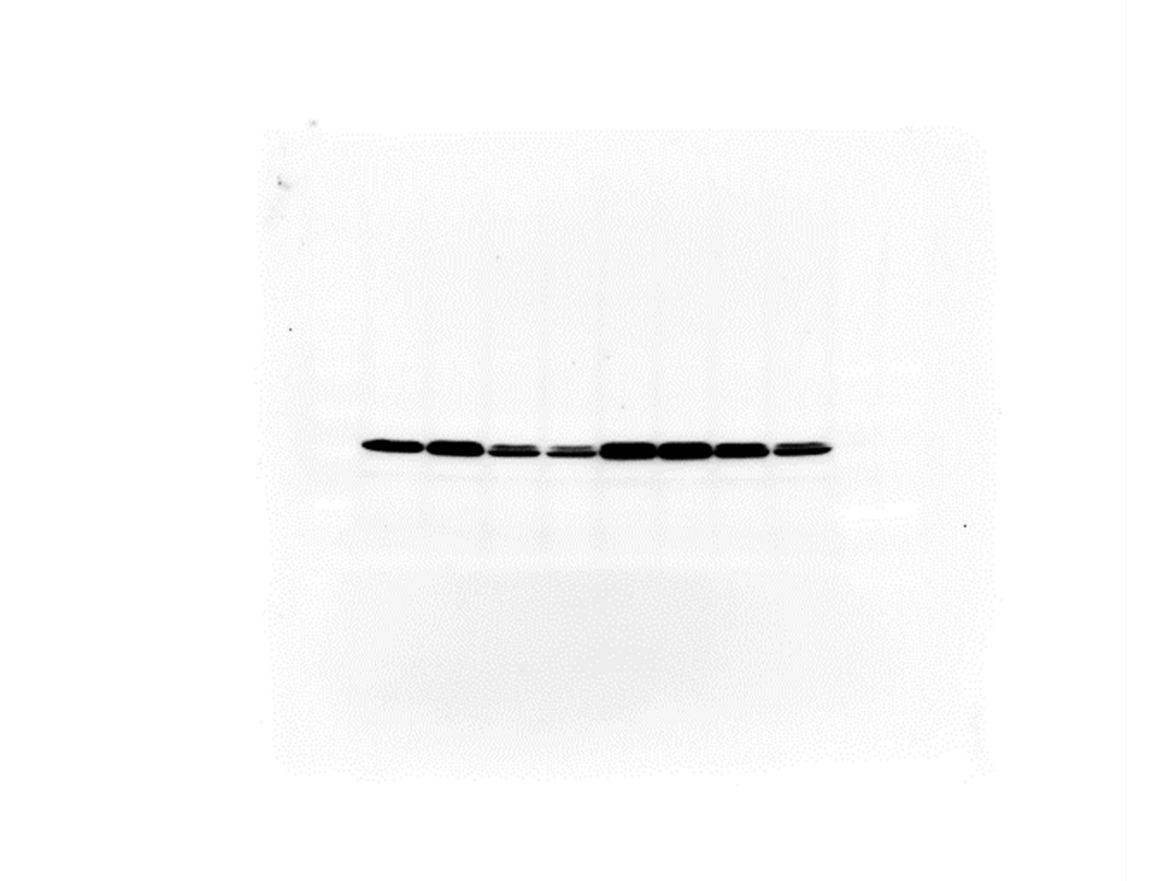
**

**Figure S9.A (pERK1/2)**


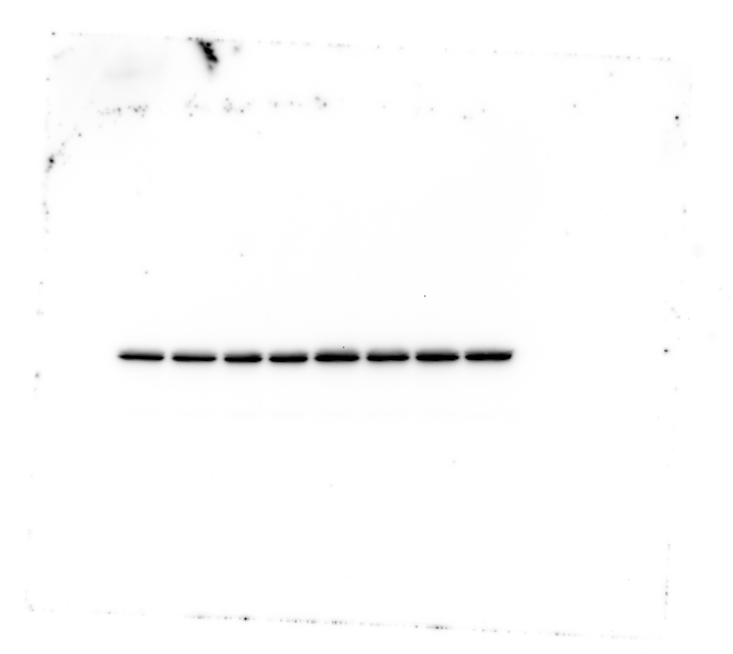


**Figure S9.A (ERK ½)**

**
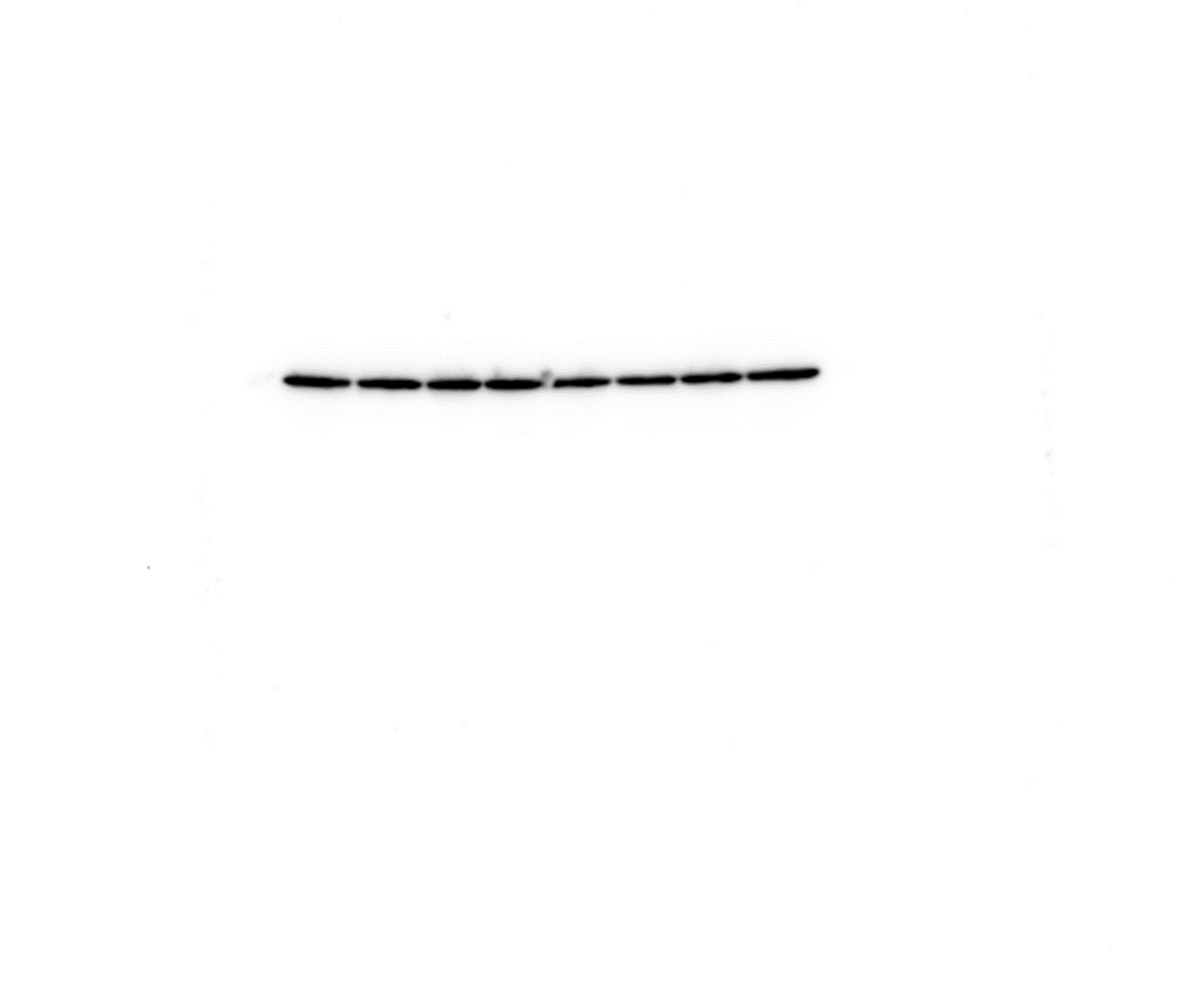
**

**Figure S9.A (GAPDH)**


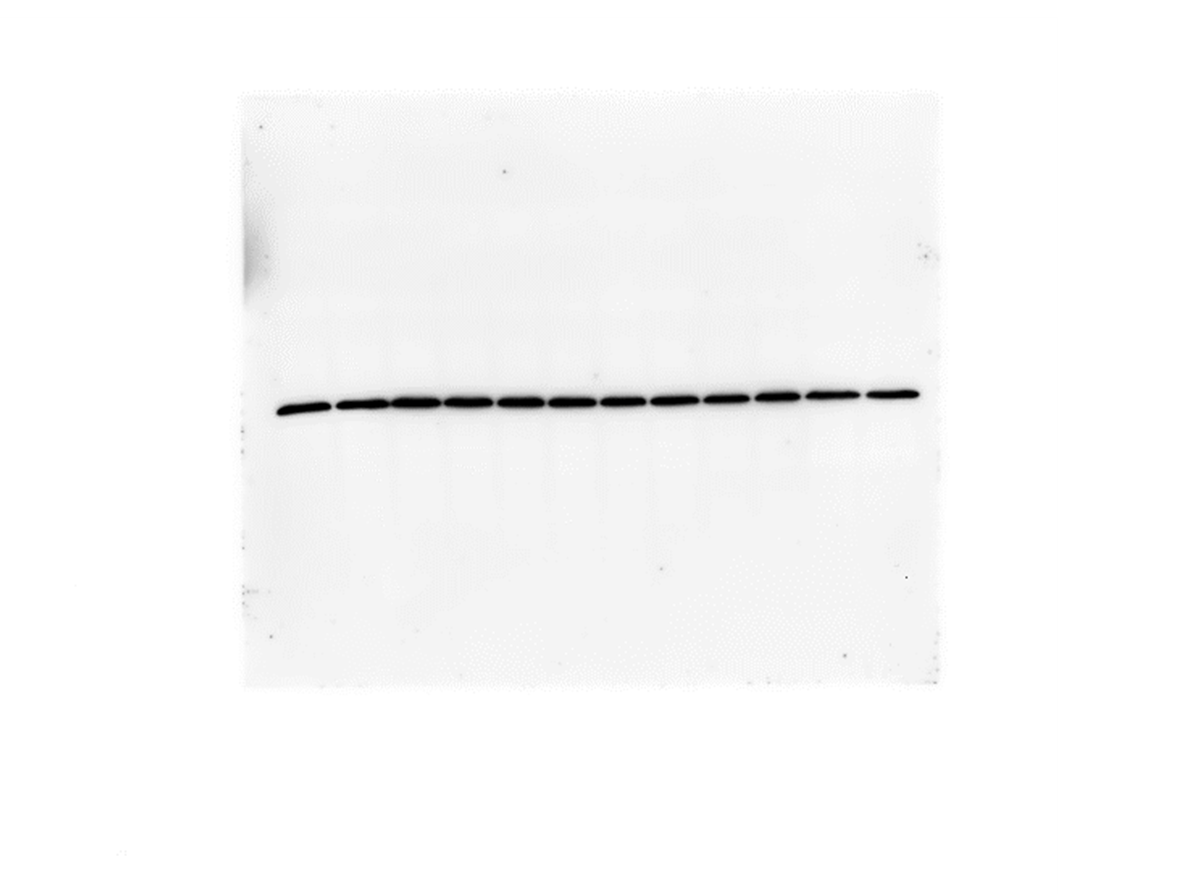


**Figure S9.B GAPDH**


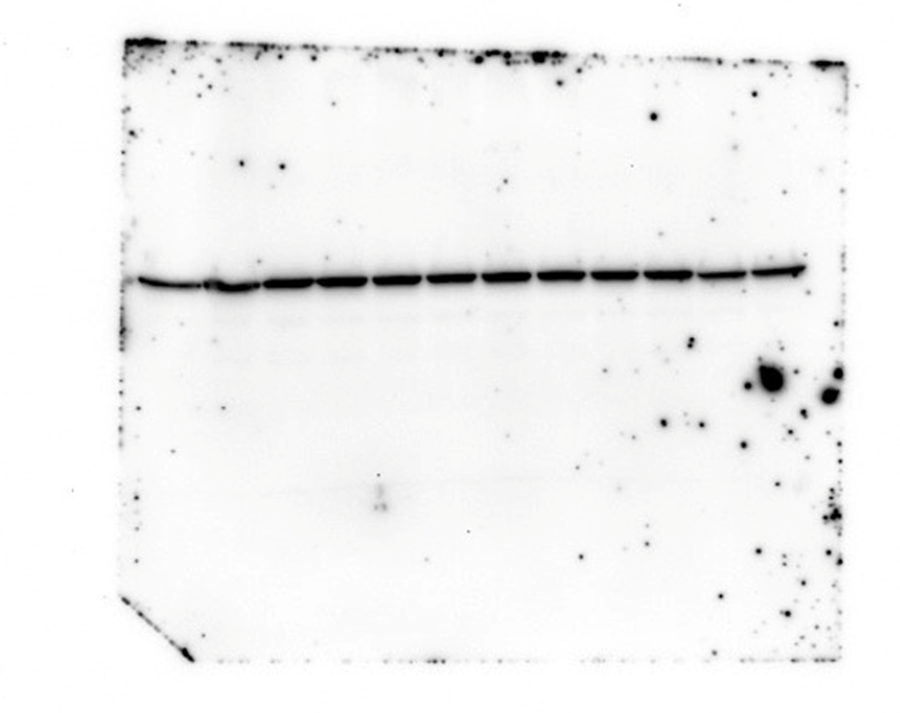


**Figure S9.B AKT**


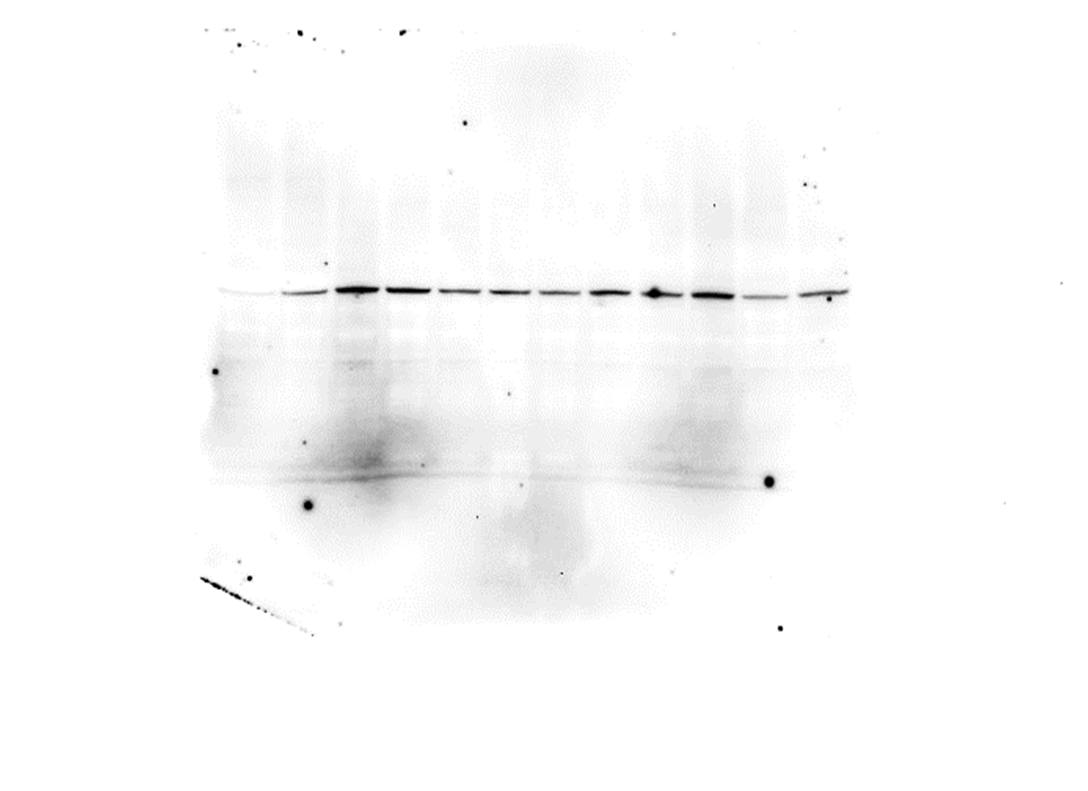


**Figure S9.B pAKT**

**
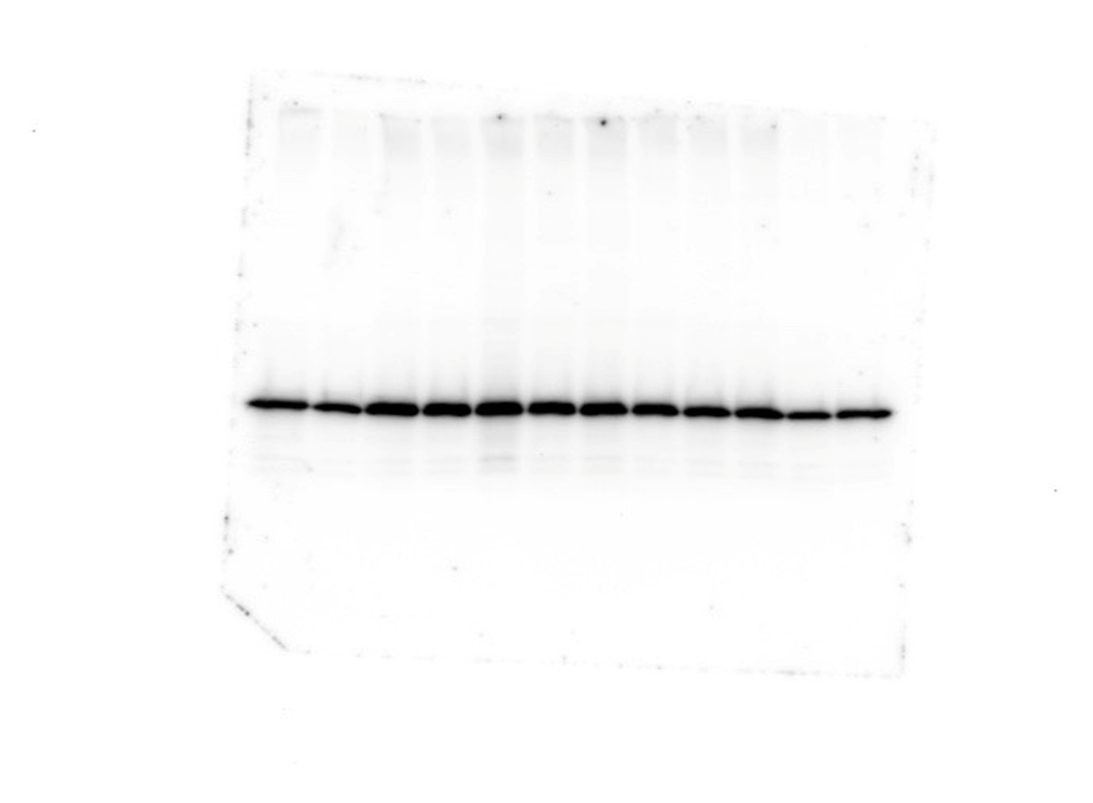
**

**Figure S9.B (S6)**

**
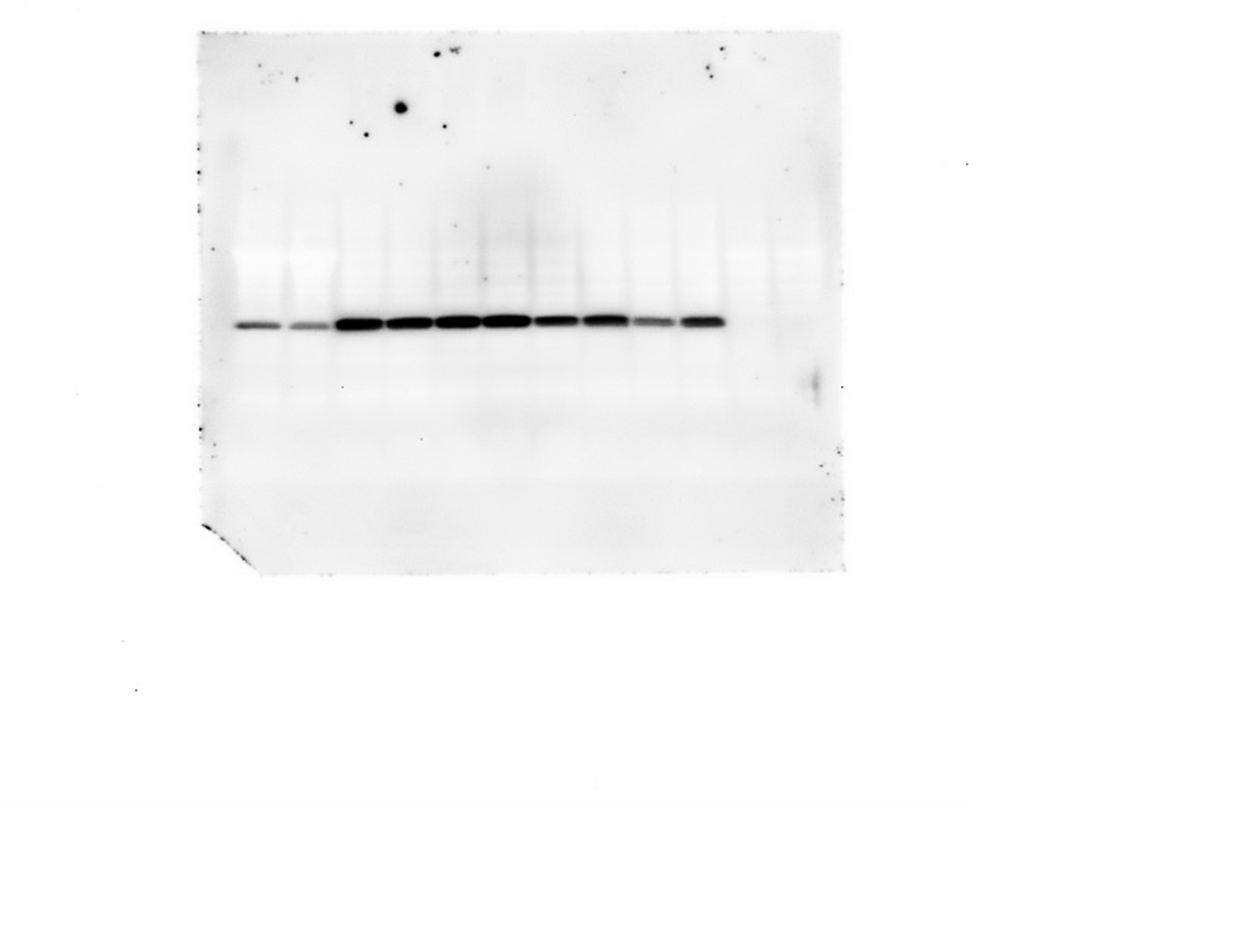
**

**Figure S9.B (pS6)**


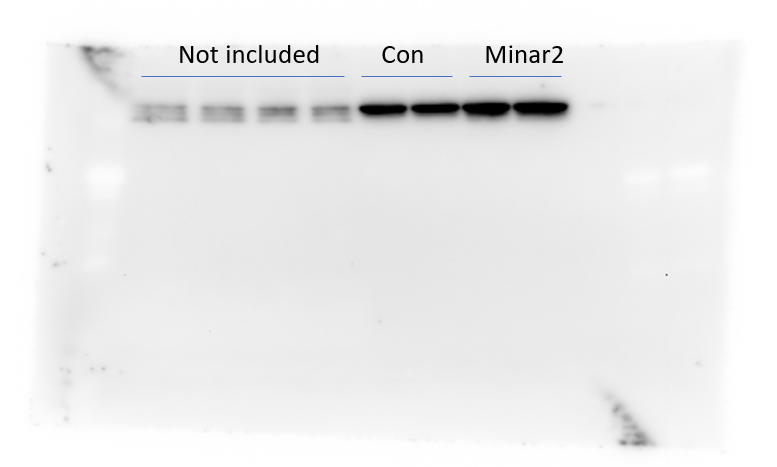


**Figure 8.C (Beta Actin)**


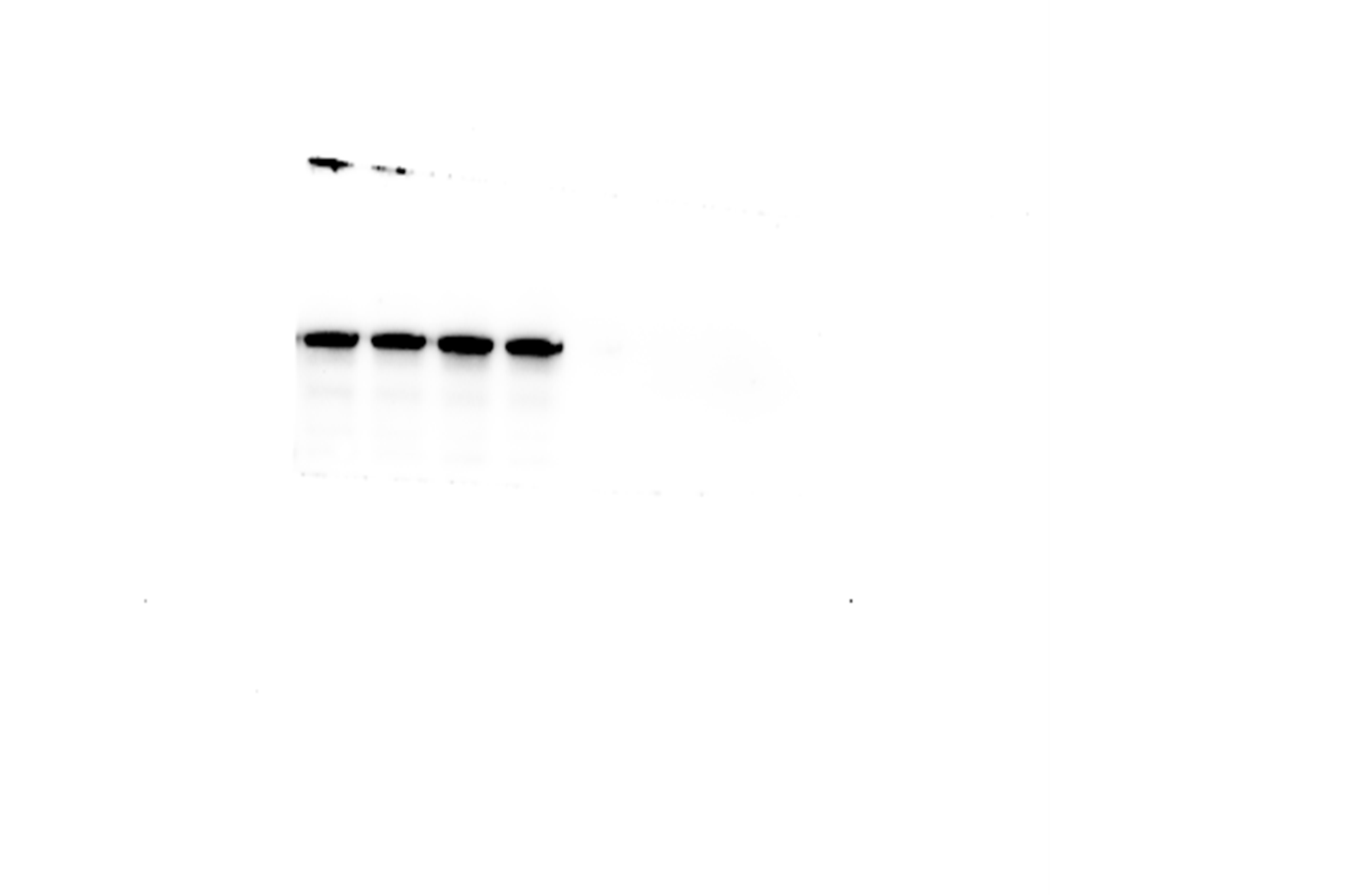


**Figure 8.C (NOTCH2)**


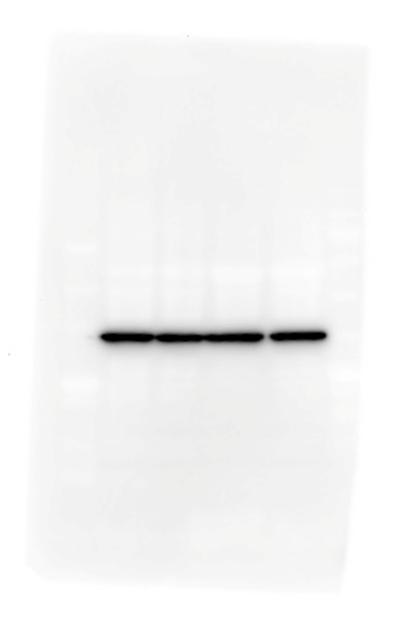


**Figure 8.B (GAPDH)**


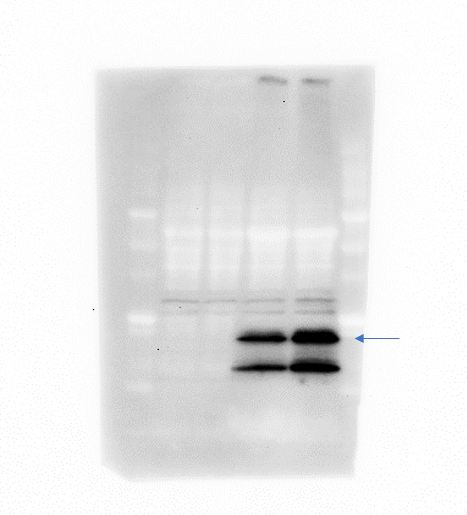


**Figure 8.B (MINAR2)**

**
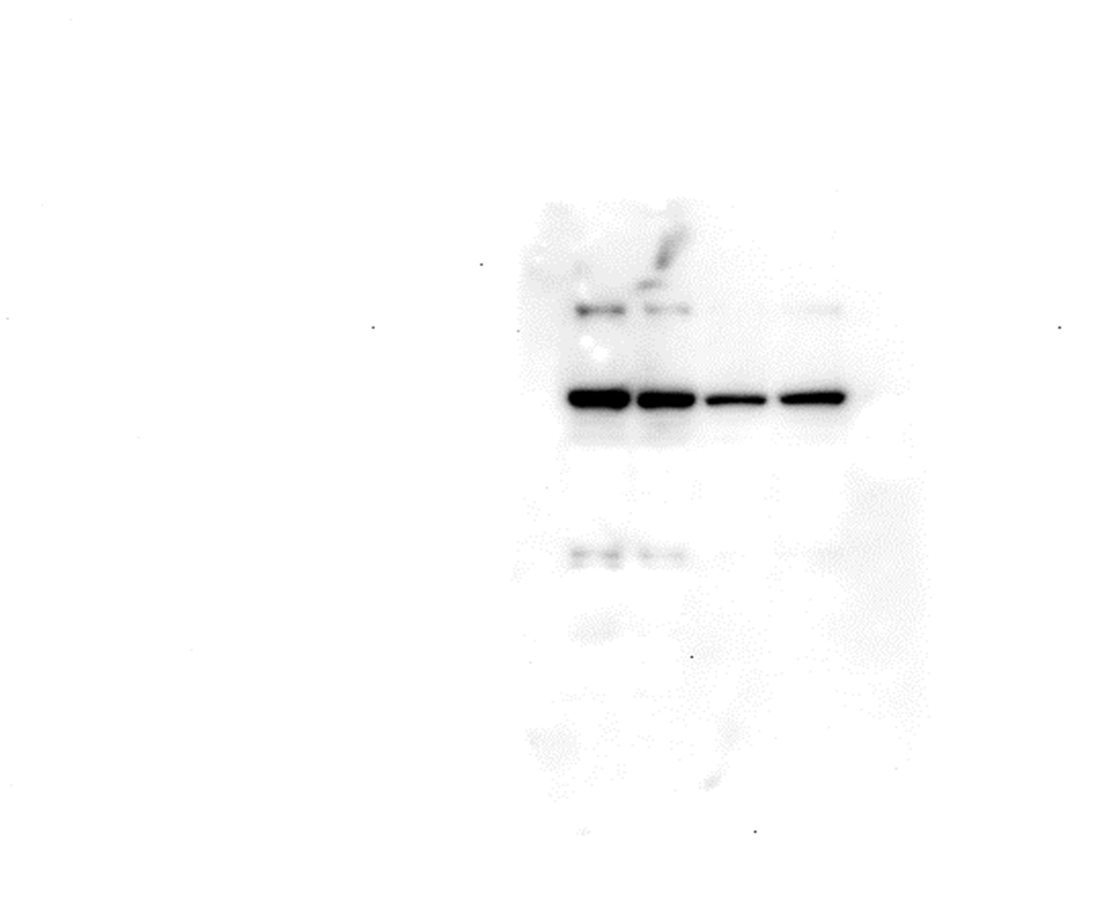
**

**Figure 8.B (NOTCH2)**


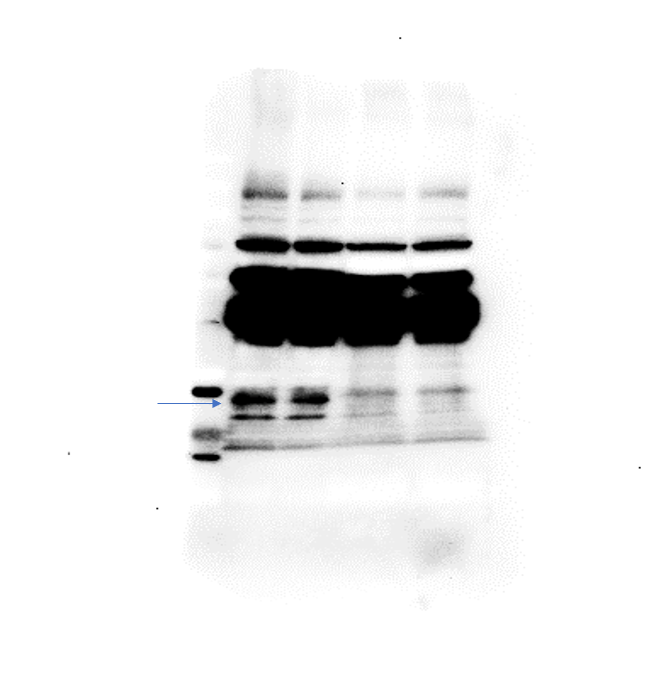


**Figure 8.B (VEGFA)**
